# Brain patterns and risk factors in the FINGER RCT multimodal lifestyle intervention

**DOI:** 10.1016/j.tjpad.2025.100390

**Published:** 2025-09-24

**Authors:** Giulia Lorenzon, Anna Marseglia, Rosaleena Mohanty, Jenni Lehtisalo, Konstantinos Poulakis, Tiia Ngandu, Alina Solomon, Miia Kivipelto, Eric Westman

**Affiliations:** a Division of Clinical Geriatrics, Center for Alzheimer Research, Department of Neurobiology, Care Sciences and Society, Karolinska Institutet, 141 52 Huddinge, Sweden; b Lifestyles and Living Environments Unit, Department of Public Health, Finnish Institute for Health and Welfare, 00300 Helsinki, Finland; c Department of Neurology, Institute of Clinical Medicine, University of Eastern Finland, FI-70211 Kuopio University Hospital, Finland; d Theme Inflammation and Aging, Karolinska University Hospital, 141 57 Huddinge, Sweden; e Institute of Public Health and Clinical Nutrition, University of Eastern Finland, FI-70211 Kuopio, Finland; f Ageing Epidemiology Research Unit, School of Public Health, Imperial College London, W6 8RP London, UK

**Keywords:** Aging, MRI, Heterogeneity, Prevention, Risk factors

## Abstract

**Importance:**

Despite the emergence of anti-amyloid therapies for Alzheimer's disease, targeting modifiable risk factors remains the most effective primary prevention strategy for dementia. While cognitive benefits of multimodal lifestyle interventions have been demonstrated, the underlying effects on brain structure remain unclear, likely due to heterogeneity in brain structure among at-risk individuals.

**Objective:**

To investigate how distinct subgroups of at-risk individuals, defined by cortical and subcortical grey matter (GM) patterns, differ in their response to the FINGER intervention, as well as in their demographic, vascular, and lifestyle profiles.

**Design:**

Observational study employing unsupervised clustering of MRI-based cortical thickness and subcortical volume metrics, followed by longitudinal assessment of a lifestyle intervention.

**Setting:**

The FINGER randomized controlled trial (RCT), a population-based, multidomain lifestyle intervention targeting older adults (aged 60–77) with elevated cardiovascular risk (CAIDE score ≥ 6) and average to slightly below-average cognitive performance.

**Participants:**

A total of 120 participants (61 intervention, 59 control) with available baseline MRI data.

**Intervention:**

Participants were randomly assigned (1:1, double-blind) to a 2-year multidomain lifestyle intervention group – targeting diet, physical activity, cognitive training, social engagement, and metabolic and vascular risk management – or to a control group receiving standard health advice.

**Main Outcomes and Measures:**

Sociodemographic, vascular, and lifestyle factors, medical comorbidities, and cognitive performance, were assessed at baseline (pre-intervention). Additionally, brain structural outcomes (mean cortical thickness, Alzheimer’s disease and resilience-related cortical signatures, hippocampal volume), and cognition (global, executive function, processing speed, memory) were analysed post-intervention using hierarchical linear models stratified by GM cluster.

**Results:**

Clusters with diffuse or frontal-predominant cortical thinning, but with more favourable vascular profiles, characterized by lower blood pressure and reduced obesity, showed significantly less cortical thinning (mean thickness, AD-signature, and resilience-signature regions; all p < 0.05) following the intervention.

**Conclusions and Relevance:**

Stratifying at-risk individuals by GM patterns and vascular risk revealed differential brain responses to the FINGER intervention. These findings underscore the value of brain-based subtyping to optimize personalized dementia prevention strategies in heterogeneous at-risk populations.

**Trial Registration:**

ClinicalTrials.gov Identifier: NCT01041989

## 1 Introduction

The prevalence of dementia worldwide is estimated to triple by 2050 [1]. While pharmacological solutions continue to be investigated [2], prevention remains the most effective strategy [1,3]. Despite the number of dementia cases having increased due to longer lifespan, age-specific incidence is decreasing in high-income countries [4]. This highlights the importance of prevention strategies even in those with a genetic predisposition for dementia. It is estimated that 14 lifelong risk factors contribute to nearly half of the dementia risk (45 %), with cardiovascular conditions and related lifestyle factors as major contributors [4]. Cardiovascular factors, including hypertension, low-density cholesterol, obesity, diabetes, alcohol consumption, smoking, and physical inactivity, also account for 32 % of all deaths [5], yet are largely modifiable [4].

The Cardiovascular Risk Factors, Aging, and Dementia (CAIDE) score was developed as a reliable tool to predict late-life dementia risk based on factors such as age, sex, education, systolic blood pressure, body mass index (BMI), total cholesterol, and physical activity [6,7]. This risk score has been used to identify individuals at higher risk of dementia for participation in the Finnish Geriatric Intervention Study to Prevent Cognitive Impairment and Disability (FINGER) multimodal intervention trial [8]. While most previous single-domain intervention trials targeting risk factors individually have failed [9,10], effective prevention trials for cardiovascular conditions have highlighted the importance of multidomain approaches [4]. The FINGER trial represents the first successful large-scale and long-term multidomain prevention trial for dementia, showing promising results in maintaining cognition after two years of intervention [11]. One previous study reported non-significant effects of the FINGER intervention on brain MRI outcomes [12]. However, detecting significant intervention-driven brain effects can be challenging due to inherent brain heterogeneity within assigned groups, as seen in pharmacological trials [13]. Subgroups based on distinct brain grey matter (GM) patterns have been demonstrated in both clinical populations, such as those with Alzheimer's, Parkinson's, Lewy-Body dementia [[14], [15], [16], [17]], and in dementia-free individuals [18]. The patterns were characterized by different cerebrovascular and cognitive profiles among dementia-free individuals [18]. Therefore, it is important to investigate the presence and characteristics of participants’ subgroups based on distinct brain GM patterns within the FINGER cohort. This is important as some subgroups may respond differently to the intervention, which has not been investigated so far.

Hence, the aims of this study are to (i) identify subgroups (clusters) of participants based on distinct cortical and subcortical GM patterns using unsupervised clustering analysis within at-risk-individuals from the FINGER multidomain Randomized Clinical Trial (RCT), (ii) examine how these GM-based clusters of participants are characterized by sociodemographic, vascular, lifestyle, medical, and cognitive factors, and (iii) investigate how these GM-based clusters of participants respond to intervention. Discovering and characterizing individuals who benefit more versus those who benefit less is of great importance, thus maximizing FINGER's effectiveness for future precision prevention strategies.

## 2 Methods

### 2.1 Study design and participants

The FINGER trial is a population-based, multidomain lifestyle intervention trial (ClinicalTrials.gov NCT01041989) targeting older adults aged 60 to 77 years who have an elevated CAIDE risk score (≥ 6) and exhibit cognitive performance that is average or slightly below average on the Consortium to Establish a Registry for Alzheimer’s Disease (CERAD) neuropsychological battery. This group is at increased risk for developing dementia [8,[19], [20], [21]]. For the full trial description and primary findings, see previous publications [11,22,23].

A total of 1260 participants were eligible and randomly assigned to either a multidomain lifestyle intervention group (*n* = 631) or a regular health advice control group (*n* = 629) in a 1:1 ratio through double-blind randomization [22]. The multidomain intervention comprised diet, exercise, cognitive training, social stimulation, and management of metabolic and vascular risk, all supervised by professionals following established guidelines and trial intervention protocol. The control group received standard health advice as per usual practice [23].

**Fig. 1:**
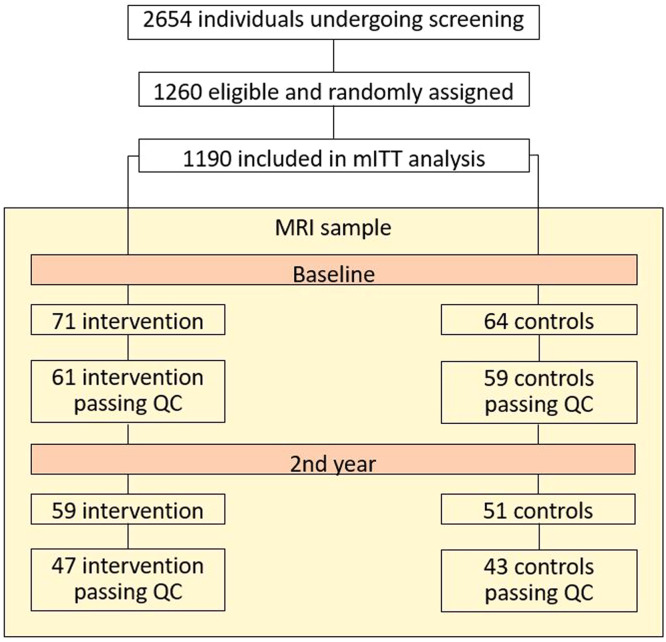
Flowchart of the MRI sub-sample from FINGER. Participants were drawn from the FINGER intervention trial (ClinicalTrials.gov NCT01041989) with available MRI scan. The MRI sub-sample after image quality control (QC) included 120 participants with scan at baseline (61 intervention and 59 controls), and 90 participants with repeated scan at 2-year follow-up (47 intervention and 43 controls). The number of excluded scans due to poor image quality slightly differ from the original RCT flowchart due to newly performed visual quality control with the newest FreeSurfer version in this study. mITT=modified intention-to-treat.

A total of 135 participants underwent Magnetic Resonance Imaging (MRI) assessment at baseline, of which 110 had a repeated scan at the 2-year follow-up (15 MRI failed FreeSurfer processing at baseline and 12 at the 2-year follow-up). This resulted in a final sample of 120 individuals at baseline (61 intervention and 59 controls) and 90 with repeated scans at both baseline and 2-year follow-up (47 intervention and 43 controls) for the present study. The study flowchart is presented as Fig. 1. The trial received approval from the Coordinating Ethics Committee of the Helsinki and Uusimaa Hospital District, and all participants provided written informed consent. Participants in the FINGER MRI sub-study gave a separate consent for MRI scans [12].

### 2.2 Sociodemographic, vascular, lifestyle, and medical information

Information on sociodemographic (age, sex, education, marital status), lifestyle (smoking, dietary habits including alcohol consumption, physical activity), and medical comorbidities (hypertension, hypercholesterolemia, diabetes, myocardial infarction, and stroke) were acquired through self-reported structured questionnaires. Details on data acquisition have been reported elsewhere [22]. Additionally. the baseline study visit included a physical examination conducted by trained physicians, as well as measurements of height, weight, waist and hip circumference, and systolic and diastolic blood pressure performed by trained nurses. Venous blood samples were collected 2 h after 75 g glucose oral administration in the participants without history of diabetes. Total serum cholesterol and plasma glucose concentrations were measured enzymatically using commercial reagents on a clinical chemistry analyser (Abbott Laboratories, Abbott Park, IL, USA).

### 2.3 Cognitive outcomes

A thorough cognitive assessment using a set of standard neuropsychological tests was administered by trained psychologists. Four cognitive outcomes were included. The primary cognitive outcome was pre-/post-intervention change in the neuropsychological test battery (NTB), calculated as a composite score based on the results from 14 tests (Z scores standardized to the baseline mean and SD, with higher scores indicating better performance). The executive function domain included Category Fluency Test, Digit Span, Concept Shifting Test (condition C), Trail Making Test (shifting score B-A), and a shortened 40-stimulus version of the original Stroop test (interference score 3–2). The processing speed domain included Letter Digit Substitution Test, Concept Shifting Test (condition A), and Stroop test (condition 2). The memory domain included Visual Paired Associates immediate (score range, 0–18) and delayed (score range, 0–6); Logical Memory immediate (score range, 0–25) and delayed (score range, 0–25) of the Wechsler Memory Scale-Revised (WMS-R); and Word List Learning (score range, 0–30) and Delayed Recall (score range, 0–10) of the CERAD test battery. Details on data acquisition have been reported elsewhere [22].

### 2.4 Brain outcomes

MRIs were acquired in three sites, in Turku using 3T Ingenuity, Philips (3D TFE sequence, voxel size 1.0 × 1.0 × 1.0 mm3, TR 8.1 ms, TE 3.7 ms), in Kuopio and Oulu using 1.5T Avanto, Siemens (3D-MPRAGE sequence, voxel size 1.2 × 1.2 × 1.2 mm3, TR 2400 ms, TE 3.5 ms, TI 1000 ms). Each site used the same imaging protocols at baseline and 2-year follow-up [12].

***Pre-processing*.** All T1-weighted images were pre-processed automatically using FreeSurfer 7.3.2 and managed through TheHiveDB database system at Karolinska Institutet, Sweden [24]. Cortical thickness measurements from both hemispheres were obtained for a total of 34 cortical regions of interest (ROIs) using the Desikan atlas [25]. Additionally, volume measurements from both hemispheres were extracted for 7 subcortical ROIs (hippocampus, thalamus, amygdala, putamen, globus pallidus, nucleus accumbens, caudate nucleus). All cortical thickness and volumetric ROIs from left and right hemispheres were averaged. A measure of intracranial volume (ICV) was also extracted from FreeSurfer.

***Quality control*.** Visual quality control (QC) was performed in-house for every brain scan (*n* = 245), to control for normalization, registration or segmentation errors during cross-sectional and longitudinal pre-processing [26]. We excluded 27 images (11 %) due to image abnormalities and/or poor quality. Additional Principal Component Analysis was run to spot outliers with abnormal regional or total intracranial volumes.

***Multi-site harmonization*.** Cortical thickness and subcortical volume measures from cross-sectional MRI scans were harmonized using ComBat, a batch-effect correction toolbox calculating and removing inter-scanner variability across the three sites while preserving biological variability [27]. For this correction, each MRI scanner was treated as a separate batch, resulting in four batches from the FINGER cohort, two 1.5T Siemens scanners in Kuopio and Oulu, and two different 3T Philips scanners in Turku. A design matrix was added to preserve effects of age and gender while removing scanner effects in the ComBat correction. Pre-post ComBat harmonization comparison revealed significant reduction of systematic scanner-related differences, while age- and gender-related variations were unaltered. Both hemispheres were similarly harmonized.

### 2.5 Statistical analysis

***Identification of baseline brain patterns*.** All statistical analyses were performed with the R software version 4.0.3. We included 41 regions of interest (ROIs), 34 cortical thickness and 7 subcortical volumes. Prior clustering, subcortical regions were corrected for ICV using residuals from least-squares linear regression between each volume and ICV [28]. Subsequently, to identify the clusters of participants based on their GM patterns, we performed unsupervised clustering on all participants’ Freesurfer output (41 ROIs) using the random forest algorithm (randomForest package, version 4.7–1.1), which was previously applied to neurodegenerative disorders and healthy aging elsewhere [[14], [15], [16],^18^]. Here, the optimal random forest parameters were ntree=3000, mtry=6, nodesize=12, while the optimal cluster solution was chosen based on a composite score of Dunn and Calinski-Harabasz (CH) indices [29]. Finally, we computed Cohen's d statistics through pairwise independent *t*-test for each ROI separately between the reference cluster vs. the rest of the clusters to identify the most significant ROIs driving the clustering of participants (averaged ROIs over cluster comparisons). Briefly, the reference cluster included individuals with relatively thicker GM, reflecting more preserved brain structure (see **paragraph “Identification of baseline GM-based clusters of participants”** for details). This group was used as a reference based on the assumption that individuals with greater brain integrity may have less room for structural improvement in response to the intervention as they have maximized it. False Discovery Rate (FDR) correction for multiple comparisons was applied (p value <0.05). Brain maps were corrected for age and gender.

***Characterization of the brain patterns*.** To investigate how the identified GM-based clusters of participants differed in sociodemographic, vascular, lifestyle, medical, and cognitive variables, we first performed descriptive statistics across all clusters using chi-square test for categorical variables, ANOVA for continuous normally distributed variables, and Kruskal-Wallis test for non-normally distributed variables. Then, we calculated Odds Ratios (ORs) and 95 % confidence intervals (CIs) through Multinomial Logistic Regression (MLR, nnet package, version 7.3–19), with the reference cluster. We fitted four separate MLR models: (1) *Sociodemographic characteristics*, including age, gender, years of education, marital status (married/cohabiting vs. living alone); (2) *Vascular risk factors* included systolic and diastolic blood pressure (mm/Hg), serum total cholesterol (mmol/L), 2-h oral glucose tolerance test (mmol/L), and BMI (kg/m^2^), all standardized per 1 standard deviation change; (3) *Lifestyle*, proxied by a composite score including physical activity two or more times per week, current smoking, alcohol consumption once per week or more, fish intake at least twice per week, and vegetable intake at least once per day; (4) *Self-reported medical disorders*, divided into cardiovascular (history of hypertension, myocardial infarction or cerebrovascular events in the last year) and metabolic disorders (history of hypercholesterolaemia or diabetes).

Additionally, we evaluated whether GM patterns predicted cognitive status through four separate Generalized Linear Models (GLMs) with the standardized cognitive scores as outcomes (global cognition, executive functioning, processing speed, and memory) and cluster allocation as the predictor. The risk of multicollinearity was assessed through Person’s correlation tests between biologically related variables. A two-sided *p* value <0.05 indicated statistical significance.

***Longitudinal evaluation of the intervention effects*.** We selected participants with both baseline and 2-year follow-up observations available (*n* = 90). In the overall FINGER sample, 28 % of the participants showed cognitive decline in total cognition (NTB) [11]. To examine the effects of time, randomization group, and GM-based participant clusters on cognitive and brain outcomes, we conducted a series of hierarchical linear models using *hlme* function from the *lcmm* R package, version 0.6.0, to accommodate more complex hierarchical structures. Time was modelled as a continuous variable using age, allowing us to capture developmental trajectories. Cognitive outcomes included global cognition, executive functioning, processing speed, and memory. Brain outcomes comprised four structural measures: mean cortical thickness (as a marker of nonspecific neurodegeneration) [30]; an AD-specific cortical signature, calculated as the mean thickness of the bilateral entorhinal, inferior temporal, middle temporal, and fusiform regions, adjusted for cortical surface area [31]; a brain resilience signature, derived from the average thickness of the anterior cingulate and temporal pole, also adjusted for surface area [32], and hippocampal volume, adjusted for ICV [11]. All brain measures were normally distributed and analysed as continuous variables. Adjustments for age, gender, and years of education were made in all models.

***Power calculation*** Minimum detectable effects (MDEs) were first estimated using standard *t*-test approximations. For the overall sample, hierarchical mixed-effects simulations incorporating repeated measures and within-subject correlations provided realistic power estimates. For small clusters we report only MDEs, as simulated power would be misleading due to limited sample sizes.

## 3 Results

### 3.1 Basic characteristics

Table 1 shows that the MRI sub-sample (*n* = 120) is comparable to the participants in the full sample [11] (*n* = 1190) with respect to sociodemographic, vascular, lifestyle, and medical baseline characteristics, confirming the representativeness of the MRI sample employed in this observational study. There were no significant differences between the intervention and control group in the MRI sample.

**Table 1:** Baseline characteristics of participants in the total sample [11] vs the MRI sample.

	Total sample (n = 1190)			MRI sample (n = 120)		
	Participants with information available	Intervention group (*n* = 591)	Control group (*n* = 599)	Participants with information available	Intervention group (*n* = 61)	Control group (*n* = 59)
**Sociodemographic characteristics**						
Age at the baseline visit, years	1190	69.5 ± 4.6	69.2 (4.7)	120	70.3 ± 4.8	69.6 (4.5)
Number of women	1190	267 (45 %)	284 (47 %)	120	27 (44.3 %)	32 (54.2 %)
Education, years	1179	10.0 ± 3.4	10.0 (3.4)	120	9.4 ± 2.5	9.2 (2.7)
Married or cohabiting	1189	436 (74 %)	454 (76 %)	119	49 (80.3 %)	46 (79.3 %)
**Vascular factors**						
Systolic blood pressure, mm Hg	1179	140.1 ± 16.7	139.8 (15.7)	120	138.2 ± 15.6	137.9 (13.7)
Diastolic blood pressure, mm Hg	1179	80.5 ± 9.6	80.1 (9.3)	120	78.5 ± 8.6	78.8 (8.7)
Serum total cholesterol, mmol/L	1186	5.2 ± 1.0	5.2 (1.0)	120	5.0 ± 1.0	4.9 (0.9)
Fasting plasma glucose, mmol/L	1188	6.1 ± 0.8	6.1 (1.0)	120	6.1 ± 0.8	6.1 (1.0)
2 h oral glucose tolerance test, mmol/L	1031	7.0 ± 2.1	7.0 (2.2)	120	6.5 ± 1.7	6.9 (2.1)
Body-mass index, kg/m²	1179	28.3 ± 4.5	28.1 (4.9)	120	28.1 ± 3.5	27.0 (3.3)
**Lifestyle factors**						
Physical activity two or more times per week	1180	410 (70 %)	427 (72 %)	116	43 (70.5 %)	44 (80.0 %)
Current smokers	1186	58 (10 %)	48 (8 %)	112	1 (1.8 %)	3 (5.4 %)
Alcohol drinking at least once per week	1182	265 (45 %)	265 (45 %)	119	34 (55.7 %)	32 (55.2 %)
Fish intake at least twice per week	1183	316 (54 %)	304 (51 %)	118	39 (63.9 %)	30 (52.6 %)
Daily intake of vegetables	1187	360 (61 %)	374 (63 %)	120	42 (68.9 %)	38 (64.4 %)
**Self-reported medical disorders**						
Hypertension	1177	392 (67 %)	387 (65 %)	116	42 (71.2 %)	31 (54.4 %)
Hypercholesterolaemia	1180	389 (66 %)	414 (70 %)	116	45 (76.3 %)	40 (70.2 %)
Diabetes	1180	76 (13 %)	74 (12 %)	116	7 (11.9 %)	7 (12.3 %)
History of myocardial infarction	1184	29 (5 %)	31 (5 %)	116	3 (5.1 %)	2 (3.5 %)
History of stroke	1181	32 (5 %)	34 (6 %)	115	2 (3.4 %)	1 (1.8 %)
Cognitive domains*						
NTB total score	1190	–0.03 ± 0.55	0.03 (0.59)	120	−0.08 ± 0.49	0.00 (0.54)
Executive functioning	1189	–0.03 ± 0.66	0.03 (0.69)	120	−0.02 ± 0.56	−0.05 (0.58)
Processing speed	1190	–0.02 ± 0.78	0.05 (0.84)	120	−0.09 ± 0.82	0.10 (0.75)
Memory	1190	–0.03 ± 0.68	0.03 (0.66)	120	−0.12 ± 0.57	−0.01 (0.62)

Data are presented as Mean (Standard deviations) for continuous variables or number (proportion %) for categorical variables. There were no significant differences between the intervention and control group in the overall sample as well as in the MRI sample. NTB=neuropsychological test battery.

⁎ Scores on the NTB total score, and on executive functioning, processing speed, and memory are mean values of Z scores of the cognitive tests included in each cognitive outcome, with higher scores suggesting better performance.

### 3.2 Identification of baseline GM-based clusters of participants

**Fig. 2:**
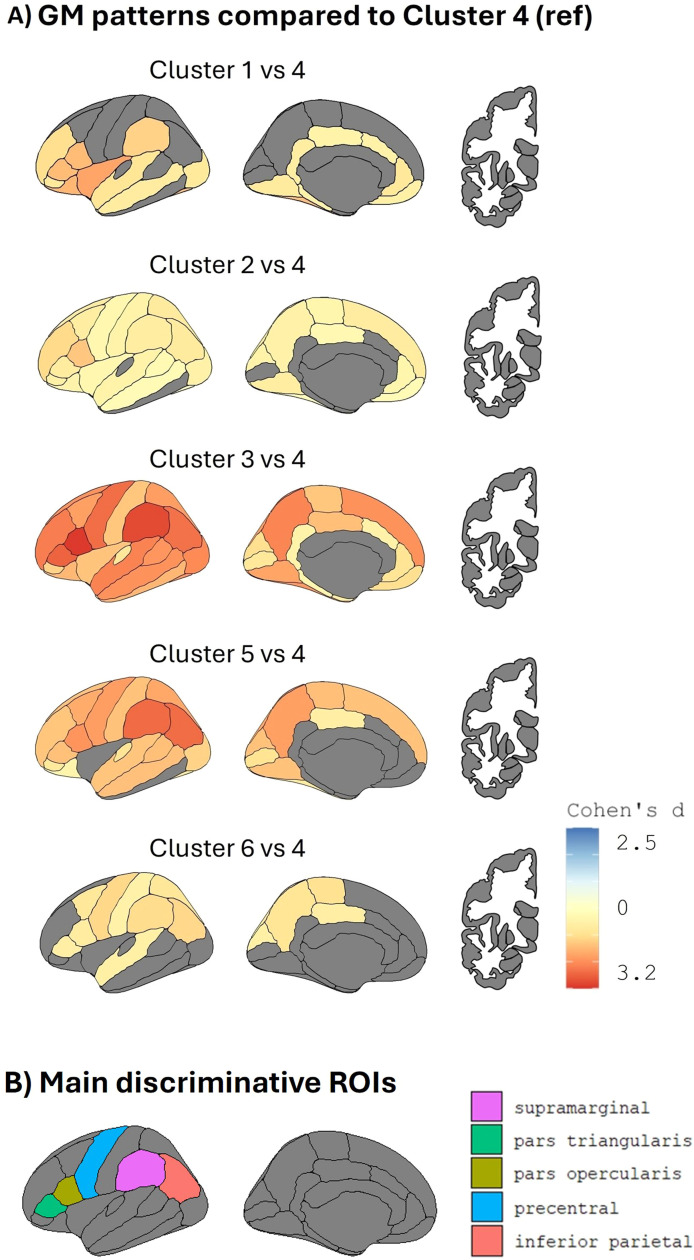
**(A)** The five identified patterns of grey matter cortical thickness (left) and subcortical volume (right) with respect to Cluster 4 (reference). Cold colours (blue/light blue) indicate greater thickness/volume, whereas hot colours (red/yellow) indicate less thickness/volume. The colour bar on the bottom-right corner denotes Cohen’s d effect sizes. Grey colour indicates non-significant difference from the reference (significant threshold *p* < 0.001). **(B)** The most discriminative brain regions separating each cluster from the reference. Right and left hemispheres are averaged. For visualization purposes, we show left lateral and medial view for cortical regions and left coronal view for subcortical regions.

**Fig. 3:**
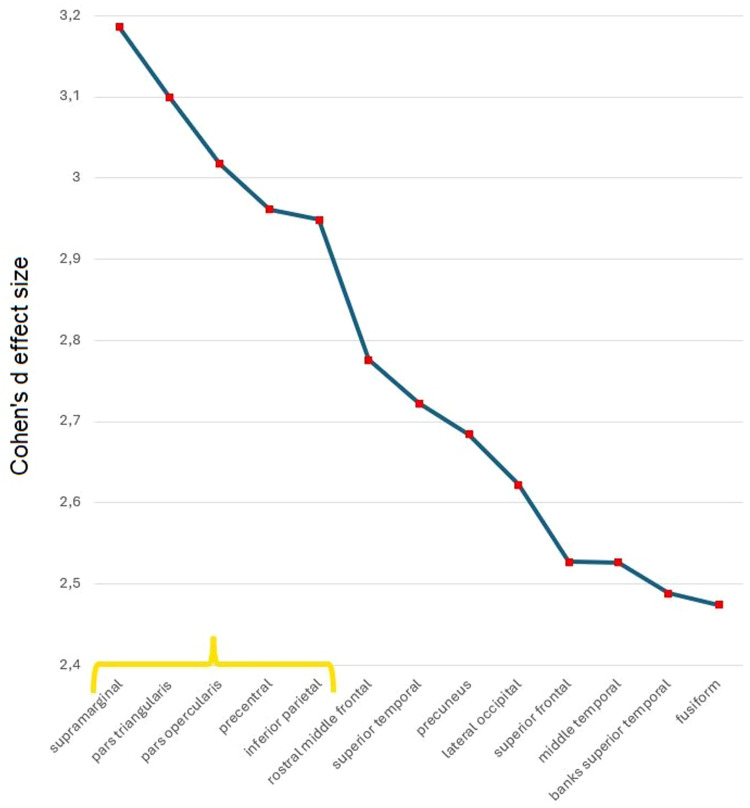
Ranked Cohen’s d effect sizes for differences in cortical thickness and volume between cluster 4 (reference) and the other clusters across all regions of interest (ROIs). The y-axis represents Cohen’s d values, indicating the magnitude of difference from the reference cluster, ordered from largest to smallest (averaged ROIs over cluster comparisons). The yellow curly bracket highlights the top ROIs selected as the major drivers of cluster differentiation, based on the visual criterion of a marked drop in effect size. For the sake of visualization, only the ROIs with large effect size are depicted here.

The optimal cluster solution included 6 clusters of participants (**Supplementary Figure 1**), Cluster 4 (*n* = 36 individuals, 30.0 % of the sample), followed by Cluster 2 (*n* = 24, 20.0 %), Cluster 3 (*n* = 20, 16.7 %), Cluster 5 (*n* = 14, 11.7 %), and finally Clusters 1 and 6 (*n* = 13 each, 10.8 %). All clusters of participants significantly differed from the overall mean in their regional GM thickness and volume with FDR-corrected p value *p* < 0.05 (**Supplementary Figure 2**). We chose Cluster 4 as a reference since it is the largest cluster, and it exhibited a more preserved GM pattern than average. Based on prior literature, we therefore did not expect a substantial intervention effect in this cluster [[33], [34], [35]]. Fig. 2**A** shows significant GM differences (<0.001) with respect to the reference (Cluster 4). Participants in Cluster 1 showed cortical thinning with maximum Cohen’s *d* = −2.3 (negative values indicating thinner cortex) in frontal regions such as the insula, lateral orbitofrontal, pars opercularis and pars triangularis cortex. Participants in Cluster 2 show some degree of cortical thinning with effect sizes up to −2 in several areas across the cortex. The most diffuse cortical thinning was observed in Cluster 3, surpassing Cohen’s *d* < −3.2 in several areas across the cortex. Participants in Cluster 5 reached similar Cohen’s d values < −3, but mostly in posterior regions such as supramarginal and inferior parietal regions. Finally, Cluster 6 exhibited a similar but milder pattern than Cluster 5, with posterior cortical thinning, in supramarginal, inferior parietal and precentral regions (effect sizes up to −1.8). Specifically, we computed Cohen’s d values for all ROIs, ranked them in order of magnitude, and reported the top contributors driving the clustering solution. Fig. 2**B** shows the ROIs with the highest Cohen’s d compared to the reference, thus the five main ROIs driving the clustering, i.e., pars opercularis, supramarginal, pars triangularis, inferior parietal, and precentral areas. Fig. 3 shows a drop in Cohen’s d values after these top five regions.

### 3.3 Characterization of GM-based clusters of participants through linear regression

**Fig. 4:**
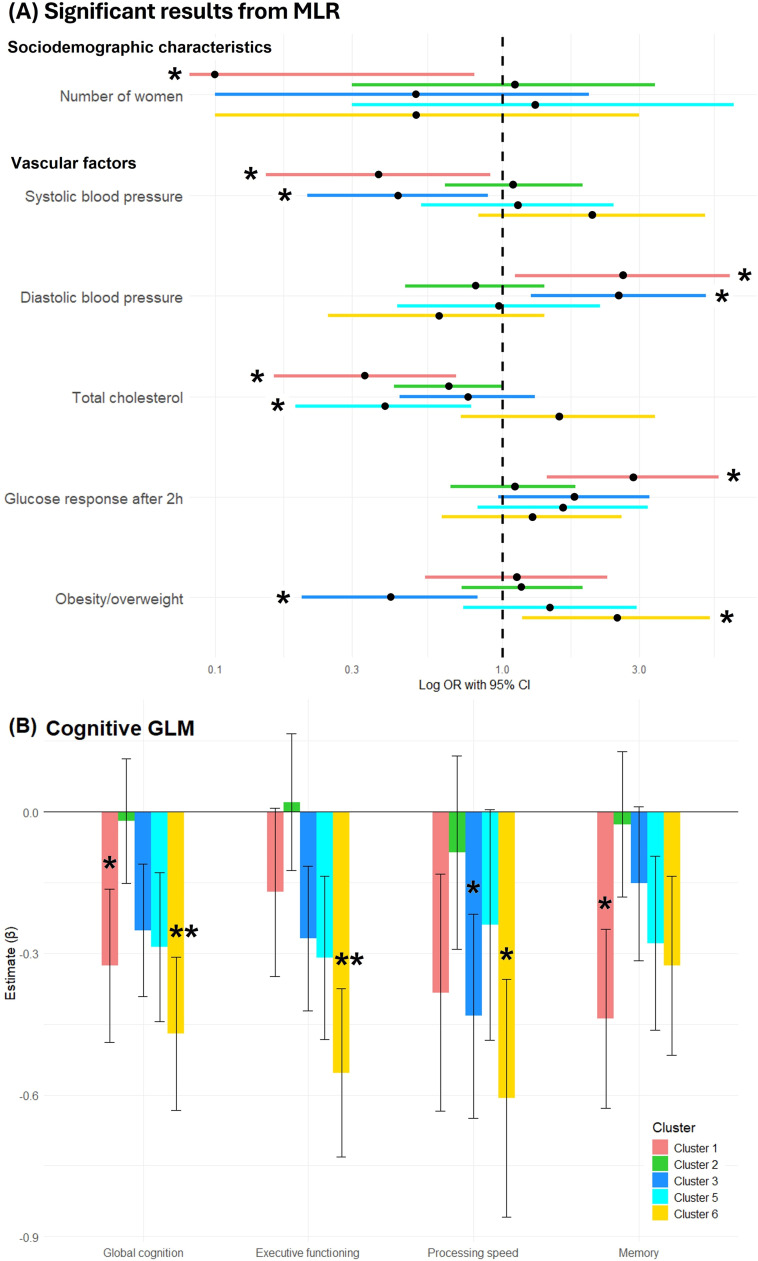
**(A)** OR and 95 % CI of the significant associations between the five GM-based clusters of participants and sociodemographic and vascular factors, compared to the reference (Cluster 4). Odds Ratios (ORs) were log transformed for visualization purposes. Black dots indicate the OR and lines in colour indicate the Confidence Intervals (CIs) of each cluster for each predictor. **(B)** Bar plot displaying the β estimates from the cognitive GLMs across the five GM-based participant clusters. Estimates reflect each cluster’s association with cognitive outcomes relative to the reference group (Cluster 4). Asterisks (*) indicate statistical significance.

**Supplementary Table 1** summarizes the descriptive characteristics of the identified clusters. Compared to Cluster 4, the multivariate MLR model (Fig. 4**A**) for sociodemographic characteristics revealed decreased prevalence of women in Cluster 1 (OR=0.1, 95 % CI [0.0, 0.8]). Clusters 1′s and 3 participants had lower systolic (OR=0.4, 95 % CI [0.2, 0.9] and OR=0.4, 95 % CI [0.2, 0.9], respectively) and higher diastolic blood pressure (OR=2.6, 95 % CI [1.1, 6.2] and OR=2.5, 95 % CI [1.3, 5.1], respectively). Cluster 1 and 5 participants showed lower total cholesterol (OR=0.3, 95 % CI [0.2, 0.7] and OR=0.4, 95 % CI [0.2, 0.8], respectively), while only Cluster 1 participants exhibited higher glucose response 2 h after intake (OR=2.9, 95 % CI [1.4, 5.7]. Finally, Cluster 3 participants had lower BMI (OR=0.4, 95 % CI [0.2, 0.8]), opposite to Cluster 6 (OR=2.5, 95 % CI [1.2, 5.3]). No significant differences were found in the lifestyle and self-reported medical conditions models (see **Supplementary Table 2** for all models).

Fig. 4**B** shows the results from the cognitive GLMs compared to the reference cluster. As expected, compared to the most preserved Cluster 4 (reference), all clusters showed lower baseline cognitive performance. Cluster 1 participants had significantly lower global cognition (β = −0.33, *p* = 0.047) and memory (β = −0.44, *p* = 0.023). Cluster 3 exhibited lower processing speed (β = −0.43, *p* = 0.048). Finally, Cluster 6 had lower global cognition (β = −0.47, *p* = 0.005), executive functioning (β = −0.55, *p* = 0.002), and processing speed (β = −0.61, *p* = 0.017). See **Supplementary Table 3** for the complete cognitive models.

### 3.4 Intervention effects on the whole MRI sub-sample

Interactions between time and intervention on the MRI sub-sample overall (*n* = 120) did not reach significance in any of the cognitive or brain outcomes. However, a marginal intervention effect emerged in processing speed (*p* = 0.071). Analyses in the overall sample had an estimated power of 75 % for MRI outcomes and 78 % for cognitive outcomes.

### 3.5 Intervention effects by cluster

**Fig. 5:**
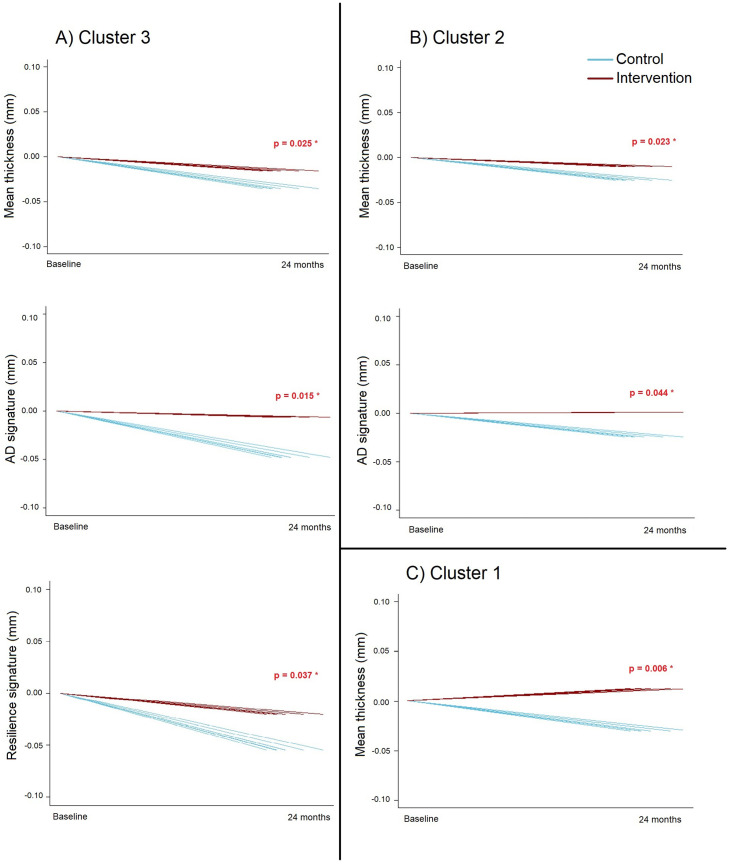
Significant intervention-driven changes from baseline to follow-up in overall mean, AD-signature, and resilience-signature thickness in **(A)** Cluster 3, **(B)** Cluster 2, and **(C)** Cluster 1. Each line represents one individual. Blue lines indicate the control group; red lines indicate the intervention group.

We found significant three-way interactions between time, randomization group, and GM-based clusters of participants. Cluster 3 participants showed less thickness decline after the intervention in terms of overall mean (β_2-years change_ = 0.05, SE = 0.02, *p* = 0.025), AD signature (β_2-years change_ = 0.07, SE = 0.03, *p* = 0.015), and resilience-signature (β_2-years change_ = 0.05, SE = 0.02, *p* = 0.037) (Fig. 5**A**). Similarly, participants belonging to Cluster 2 showed less decline in mean thickness (β_2-years change_ = 0.05, SE = 0.02, *p* = 0.023) and AD signature (β_2-years change_ = 0.05, SE = 0.03, *p* = 0.044) (Fig. 5**B**), whereas participants belonging to Cluster 1 showed less mean thickness decline (β_2-years change_ = 0.07, SE = 0.03, *p* = 0.006) (Fig. 5**C**). There were no significant interactions between time, randomization group and GM-based clusters of participants on hippocampal volume, nor in the four cognitive outcomes. Observed cortical-thickness changes in Clusters 1–3 exceeded their respective MDEs, whereas changes in Clusters 4–6 were generally below MDEs, reflecting insufficient power to detect smaller effects. Cognitive outcomes remained below cluster-specific MDEs, suggesting that subtle cognitive improvements may have gone undetected (**Supplementary Table 4**).

## 4 Discussion

This is the first study to identify distinct GM-based clusters of at-risk-individuals, characterize their sociodemographic and vascular profiles, and examine their differential response to the FINGER multimodal lifestyle intervention. Six clusters with different GM patterns and intervention response were identified. The best responders had lower GM (diffusely or frontal), but also lower cardiometabolic risk and a lower proportion of women, who are generally at higher risk for dementia [36].

In the overall study population, we found no significant differences in brain (mean thickness, AD-signature, resilience signature, hippocampal volume) or cognition (global cognition, executive functions, processing speed, memory). Unlike previous FINGER findings [11,12], we observed no cognitive benefits, likely due to the small longitudinal MRI sample (*n* = 90) [11]. However, significant intervention-by-cluster interactions revealed benefits for specific baseline GM patterns, underscoring the importance of stratified analyses for personalized prevention –key contribution of the current study.

We identified three patterns of reduced cortical thinning across the clusters: frontal (Cluster 1), diffuse (Clusters 2 and 3), and posterior (Clusters 5 and 6. Only the clusters with more diffuse or predominantly frontal cortical thinning benefitted significantly from the FINGER intervention, showing less decline over time in mean cortical thickness (Clusters 1–3), AD-signature (Clusters 2 and 3) and resilience-signature thickness (Cluster 3). Our findings show that baseline GM patterns can predict intervention response, though the predictive value may differ across disease stages. In fact, growing evidence illustrates brain heterogeneity in neurodegenerative diseases [[14], [15], [16], [17]] and aging [18] and show that a deeper phenotyping can improve detection of treatment effects [13,37]. However, in Mild Cognitive Impairment, less atrophy in AD-related regions predicted better intervention response [13]. This suggests that while thinner cortex may facilitate responsiveness in preclinical stages, plasticity limits may eventually be exceeded in later stages, reducing intervention efficacy. Also, the greater sensitivity of mean thickness as an outcome measure suggests that dementia risk in this cohort may not be predominantly AD-related [32,38,39]. Although the absence of short-term cognitive gains may limit immediate clinical interpretability, the combination of vascular protection with favourable baseline cognition in individuals responding to the intervention points to potential clinical benefits [40]. Notably, preserved brain structure may reflect slowed neurodegeneration, potentially delaying the onset or progression of impairment—referred to as “disease time saved”—, with a potential impact at the public health level. In prevention research, higher-risk individuals often show greater intervention benefits [41], reflecting baseline risk effects [42] where those with preserved brains have limited room for improvement due to ceiling effects [43,44]. Thinner cortex may also reflect neuroplasticity, supporting responsiveness through reserve mechanisms [[45], [46], [47]].

Risk and protective factors may also influence clusters’ intervention response. The most responsive clusters (showing less atrophy over time), were characterized by lower systolic blood pressure (Clusters 1 and 3), lower BMI (Cluster 3), lower cholesterol and fewer women (Cluster 1). Although the FINGER study previously found no effect of baseline vascular or sociodemographic factors on intervention outcomes [48], our stratified analyses unmasked more nuanced vascular influences [4,49,50]. Cluster 3 (diffuse lower GM) exhibited lower vascular burden (lower BMI and systolic blood pressure), as obesity is often associated with sedentary behaviour, diabetes, and hypertension, which in turn increase cardiovascular and dementia risk. Specifically, physical activity and weight loss consistently improve cognition [4], as well as elevated systolic blood pressure is associated with ∼60 % higher dementia odds [[51], [52], [53]]. Cluster 3 could therefore be considered as having a lower or better controlled vascular risk. It is however important to consider that FINGER inclusion criteria required a certain CAIDE score, therefore the absence of other elevated CAIDE risk factors in Cluster 3 could suggest underlying neurodegeneration independent from the vascular pathways. Cluster 2 (mild diffuse lower GM) also benefitted from the intervention exhibiting less mean and AD-signature atrophy over time, but lacked clear vascular or sociodemographic distinctions, raising the possibility of unidentified resilience factors. Cluster 1 participants exhibited lower frontal cortical thickness, less women [51], alongside a mixed cardiometabolic profile (lower systolic but greater diastolic blood pressure, lower cholesterol, and elevated glucose response), suggesting altered insulin regulation [54]. Cluster 1 participants response suggests they may be particularly suitable for multimodal lifestyle interventions combining diet and physical activity – the first-line treatments for older adults with prediabetes, diabetes, or hypertension [[55], [56], [57], [58], [59]]. Conversely, the non-responders (Clusters 5 and 6) were characterized by reduced posterior cortical thickness, especially in supramarginal and inferior parietal regions, implicated in naming and memory [60,61], and associated to cognitive decline [62]. Cluster 6 also had significantly elevated BMI, a possible indicator of obesity/overweight.

The main limitation of this study is the modest and uneven cluster sizes, yielding limited power to detect cognitive changes, possibly underestimating true effects. Cortical-thickness effects exceeded cluster-specific detection thresholds, whereas cognitive analyses were likely underpowered. Hierarchical modelling and FDR correction support robustness, yet small clusters results should be considered hypothesis-generating and require replication in larger, independent multidomain intervention datasets (e.g., within the World Wide FINGER network in the coming years). Additionally, the two-year follow-up may be too short for long-term cognitive benefits, emphasizing the need for extended studies. However, statistical significance does not always ensure clinical relevance, as effects may be too small to meaningfully influence health outcomes or daily functioning. As in the original FINGER trial, subgroup findings from small samples warrant caution, considering effect size, real-world impact, and prior evidence. Furthermore, restricting analyses to participants with MRI and follow-up may introduce selection bias, and the multidomain design prevents identifying specific drivers, though each component supports brain health and may act synergistically [63,64]. Finally, while we present the most discriminative regions by ranking Cohen’s d effect sizes, smaller differences across other ROIs may warrant further investigation. Despite these limitations, the FINGER study remains the only large-scale randomized trial to integrate a multidomain lifestyle intervention with longitudinal MRI and cognitive assessments, enabling detailed brain- and risk-based stratification analyses and underscoring its unique contribution to the field.

In conclusion, participants with lower cortical thickness, especially frontally or diffuse, showed the greatest brain benefit from FINGER, likely through neuroplasticity and favourable vascular profiles. Although longer follow-ups are needed to validate the clinical predictive value, these findings stress the need to account for brain heterogeneity in intervention studies. Stratifying participants by GM patterns may improve preventive strategies for high-risk groups and advance precision medicine in dementia prevention.

## Consent statement

All human subjects provided informed consent.

## Funding

Miia Kivipelto received funding from EU the Joint Programme - Neurodegenerative Disease Research (JPND) EURO-FINGERS grant; NordForsk NJ-FINGERS grant; EU Innovative Health Initiative Joint Undertaking (IHI JU) AD-RIDDLE, under grant agreement No. 101132933; Region Stockholm (ALF, Sweden); 10.13039/100012306Center for Innovative Medicine (CIMED) at 10.13039/501100004047Karolinska Institute (Sweden); Stiftelsen Stockholms sjukhem (Sweden); 10.13039/501100004063Knut and Alice Wallenberg Foundation (Sweden); and 10.13039/501100006636Swedish research council for health, working life and welfare (FORTE); Juho Vainio Foundation (Finland); Kela (Finland); Research Council of Finland.

Eric Westman was supported the Swedish Research Council (VR) No. 2016-02282, 2021-01861; the Center for Innovative Medicine (CIMED) No. FoUI-954459, FoUI-975174; the regional agreement on medical training and clinical research (ALF) between Stockholm County Council and Karolinska Institutet No. FoUI-952838, FoUI-954893; The Swedish Brain Foundation (Hjärnfonden) No. FO2022-0084, FO2024-0239; The Swedish Alzheimer's Foundation (Alzheimerfonden) No. AF-967495, AF-980387; The Swedish Parkinson's foundation (Parkinsonfonden) No. 1557/24, 1521/23; EU Innovative Health Initiative Joint Undertaking (IHI JU) AD-RIDDLE; King Gustaf V:s and Queen Victorias Foundation; Olle Engkvists Foundation (Olle Engkvists Stiftelse) No. 186-0660, 224-0069 as well as Birgitta and Sten Westerberg for additional financial support.

Anna Marseglia was supported by the Center for Innovative Medicine (CIMED) [No. FoUI-988254], the Swedish Research Council for Health, Working Life and Welfare (FORTE) [No. 2024-00210], Karolinska Institutet Research Foundation [Dnr 2024-02580], Foundation for Geriatric Diseases at Karolinska Institutet [No. 2024-02114, 2023-01598, 2022-01268], Loo och Hans Ostermans stiftelsen (No. 2024-02166, 2023-01645, 2022-01255), and stiftelsen för Gamla Tjänarinnor.

Tiia Ngandu received funding from the EU Joint Programme - Neurodegenerative Disease Research (JPND) EURO-FINGERS grant; NordForsk NJ-FINGERS grant; Research Council of Finland; Sigrid Jusélius Foundation (Finland); The Finnish Cultural Foundation; Päivikki and Sakari Sohlberg Foundation (Finland); Alzheimer’s Research and Prevention Foundation (US).

Alina Solomon received funding from the European Research Council [grant 804371]; EU Joint Programme - Neurodegenerative Disease Research (JPND) Multi-MeMo grant; EU Innovative Health Initiative Joint Undertaking (IHI JU) AD-RIDDLE, under grant agreement No. 101132933; Alzheimerfonden (Sweden); Juho Vainio Foundation (Finland); Finnish Cultural Foundation (Finland); Yrjö Jahnsson Foundation (Finland).

The sponsors had no role in the design and conduct of the study; in the collection, analysis, and interpretation of data; in the preparation of the manuscript; or in the review or approval of the manuscript.

## CRediT authorship contribution statement

**Giulia Lorenzon:** Writing – review & editing, Writing – original draft, Visualization, Validation, Software, Methodology, Investigation, Formal analysis, Data curation, Conceptualization. **Anna Marseglia:** Writing – review & editing, Supervision, Investigation, Conceptualization. **Rosaleena Mohanty:** Writing – review & editing, Visualization, Supervision, Methodology. **Jenni Lehtisalo:** Writing – review & editing, Resources, Project administration. **Konstantinos Poulakis:** Writing – review & editing, Validation, Supervision, Methodology. **Tiia Ngandu:** Writing – review & editing, Resources, Project administration. **Alina Solomon:** Writing – review & editing, Resources, Project administration, Investigation, Funding acquisition. **Miia Kivipelto:** Writing – review & editing, Resources, Project administration, Investigation, Funding acquisition, Conceptualization. **Eric Westman:** Writing – review & editing, Validation, Supervision, Resources, Project administration, Methodology, Investigation, Funding acquisition, Conceptualization.

## Declaration of competing interest

The authors declare that they have no known competing financial interests or personal relationships that could have appeared to influence the work reported in this paper.

## Appendix Supplementary materials

Image, application 1

Image, application 2

Image, application 3

Image, application 4
