# Supplementary material for: Brain patterns and risk factors in the FINGER RCT multimodal lifestyle intervention
Source: J Prev Alzheimers Dis. 2025 Sep 24;12(10):100390. doi: 10.1016/j.tjpad.2025.100390 (PMC12627893; doi:10.1016/j.tjpad.2025.100390)
Supplement: Supplementary file 2 [file mmc2.pdf]

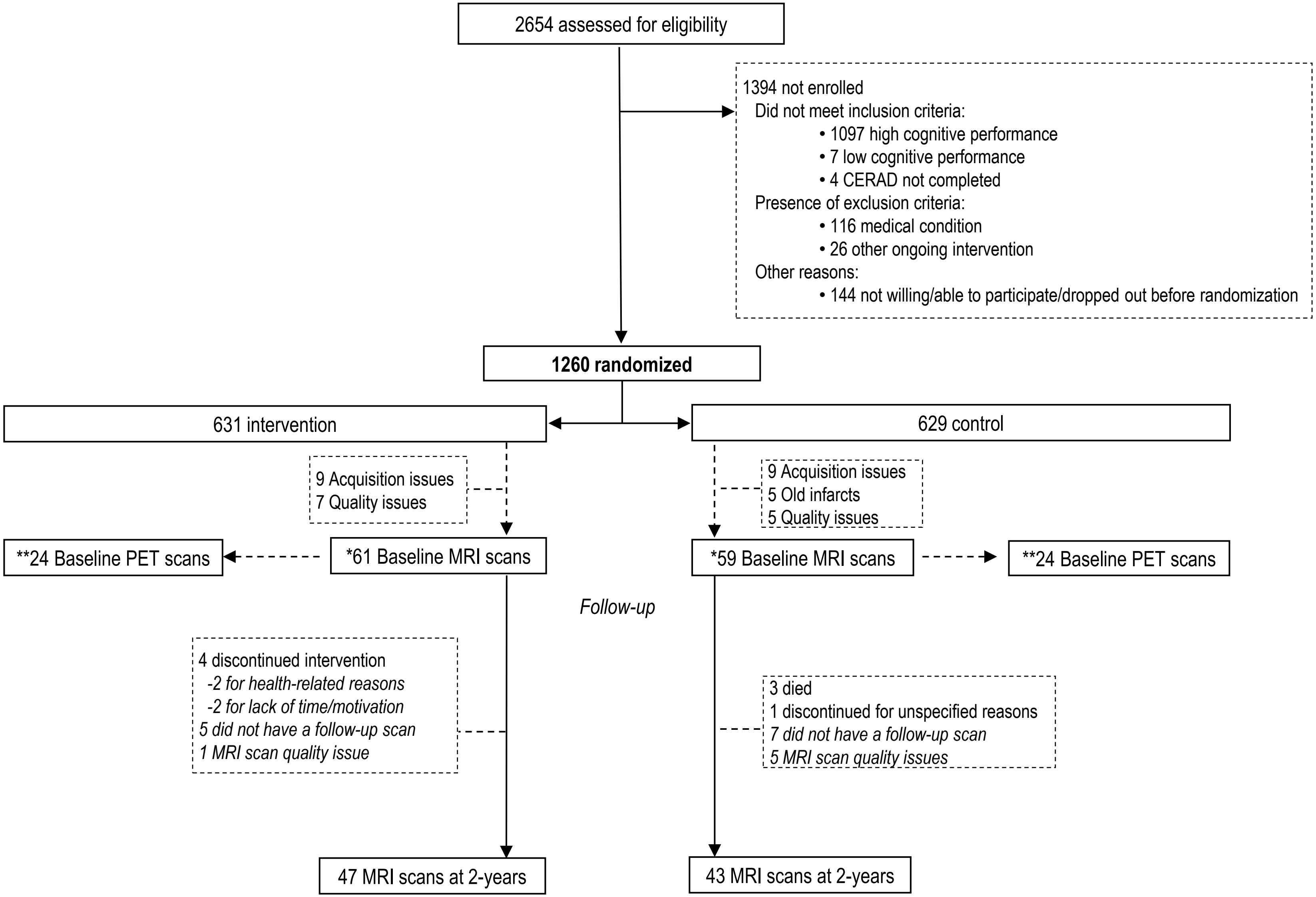

Figure 1. CONSORT Diagram Neuroimaging Sub-study in the FINGER trial

**Exploratory MRI outcome in a sub-sample at 4 trial sites (individuals (n=155) most recently recruited at the time when MRI resources became available at a specific site, and with no contraindications)*

CERAD: Consortium to Establish a Registry for Alzheimer's Disease; FINGER: Finnish Geriatric Intervention Study to prevent cognitive impairment and disability; MRI: Magnetic resonance imaging
