# Supplementary material for: Brain patterns and risk factors in the FINGER RCT multimodal lifestyle intervention
Source: J Prev Alzheimers Dis. 2025 Sep 24;12(10):100390. doi: 10.1016/j.tjpad.2025.100390 (PMC12627893; doi:10.1016/j.tjpad.2025.100390)
Supplement: Supplementary file 3 [file mmc3.pdf]

This supplement to the manuscript "*Alzheimer disease genetic risk score and neuroimaging markers in a multimodal lifestyle intervention. An exploratory analysis of a randomized controlled trial*" contains the following items:

1. Original protocol for the FINGER 2-year RCT

2. Summary of changes between the original protocol and the final published protocol for the 2-year RCT (*Kivipelto M et al, Alzheimers Dement. 2013;9(6):657-65. doi: 10.1016/j.jalz.2012.09.012*)

3. Final protocol for the full FINGER study including extended follow-up visits after the 2-year RCT (ongoing)

The manuscript "*Alzheimer disease genetic risk score and neuroimaging markers in a multimodal lifestyle intervention. An exploratory analysis of a randomized controlled trial*" describes analyses focusing on an Alzheimer disease genetic risk score (AD-GRS) in the FINGER exploratory neuroimaging sub-study. These analyses are post-hoc, i.e. the AD-GRS was published in 2022 (Bellenguez C et al, Nat Genet. 2022;54(4):412-436; doi:10.1038/s41588-022-01024-z) and was not included in the original FINGER protocol.

Original protocol (2008)

FINNISH GERIATRIC INTEVENTION STUDY TO PREVENT COGNITIVE IMPAIRMENT AND DISABILITY

RESEARCH PLAN

1. Abstract
2. Background
3. Aims and hypotheses
4. Methods
5. Relevance
6. Researchers and research environment
7. Financial aspects

1. Abstract

Dementia is a major growing cause of disability in the elderly with the numbers suffering from it proposed to quadruple by the year 2050. This will force enormous pressure on health care systems and society on the whole unless effective means for minimizing dementia incidence are introduced. Evidence from longitudinal population based studies indicates that dementia and its main subgroup Alzheimer's disease share many vascular and life-style related risk factors with cardio- and cerebrovascular diseases. A simple scoring tool, the Dementia Risk Score has recently been developed in the Cardiovascular Risk Factors, Aging and Dementia Study (CAIDE) to predict the risk of late-life dementia based on midlife risk factors. The Finnish Geriatric Intervention Study (FINGER) will plan and conduct a 2-year multi-domain intervention study aiming to lower the risk of cognitive decline in high-risk individuals. The study population of approximately 1200 individuals at an elevated risk of cognitive decline will be screened and recruited from participants of previously randomly sampled study populations. They will be randomized into two groups equal in size to receive either intensive multi-domain intervention or regular health advice. The intervention will consist of four components: nutrition, physical activity, cognitive training and social activity, and intensive monitoring and management of metabolic and vascular risk factors. The primary outcome cognitive decline will be measured using a sensitive neuropsychological test battery. Secondary outcomes to be followed are: disability, depressive symptoms, vascular risk factors and outcomes, quality of life, disability, utilization of health resources and for a subgroup changes noted on brain magnetic resonance imaging. As a two-year period is short for evaluating the slow process of cognitive decline, further follow-ups are planned to fully evaluate the effects of the intervention on the incidence of dementia and the secondary outcomes. We anticipate that this intensive multi-domain intervention on known risk factors will significantly lower the risk of cognitive impairment, postpone the incidence of dementia and have beneficial effects regarding the secondary outcomes. This consortium proposes an innovative approach combining our nations' strong expertise in epidemiological and interventional research to help resolve a major health problem of the coming decades using the scientifically valuable setting of a randomized controlled trial. Our project will provide data urgently needed for the basis of planning health services and education programs related to the prevention of dementia.

2. Background

Alzheimer's disease (AD) is the most common cause of dementia, affecting approximately 24 million people world-wide. This number is estimated to double every 20 years if no effective means of intervention are applied (1). In Finland, approximately 85 000 people have moderate to severe dementia, 35 000 have mild dementia, and 13 000 people develop dementia yearly. In the World Health Report 2003 the disability weight for dementia was higher than for most disorders, only spinal injury and cancer had higher disability weights (2). A 5-year delay in the onset of AD might half the future overall prevalence of AD (4). The key issue is in finding effective preventive or therapeutic means to delay dementia onset.

AD is a multi-factorial syndrome resulting from genetic-environmental interactions. Advanced age, familial aggregation, and the susceptibility gene apolipoprotein E (ApoE) ε4 allele have long been recognized as significant risk factors for AD. These traditional risk factors created a fatalistic view of AD in the past but recent longitudinal studies have indicated several modifiable vascular and life-style related risk factors for AD (5). The identification of modifiable risk factors points to the possibility of delaying or even preventing AD onset. As the process leading to dementia in AD takes decades (6), long-term prospective studies are required to understand the role of the natural course of the disease and its modifiable risk factors. Detailed data on these risk factors is necessary for the development of therapeutic strategies and population education and health programs aiming to prevent or delay AD onset (5). Clinical, genetic and interventional studies are also needed to clarify the pathogenic mechanisms and to translate research findings into practical guidelines to prevent and treat AD efficiently. The ApoE ε4 is relatively common in the Finnish general population (ca 30% and among the demented ca 50%), enabling exploration of effect modification by ApoE ε4 in the current project.

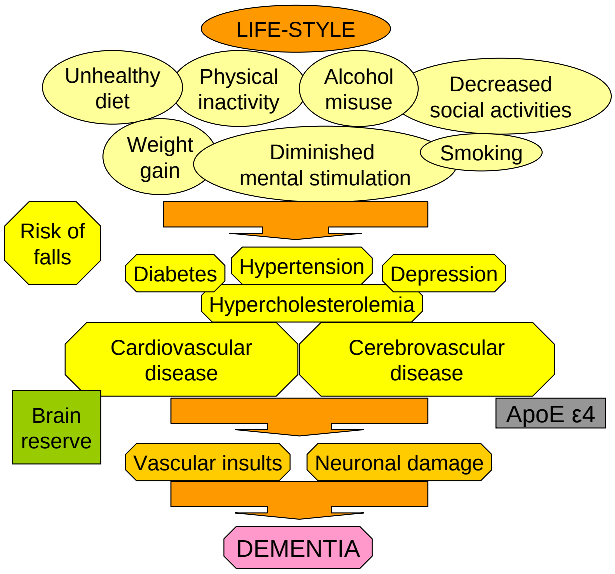

Figure 1. Possible processes and targets of intervention for dementia

a. Modifiable risk factors for dementia and AD

Based on a recent systematic literature review (studies published during 1987-2004) (PI member of the group) (7) and studies published after 2004, there is strong evidence that advanced age and the ApoE ε4 allele are the most important risk factors for dementia/AD; moderate/strong evidence that high blood pressure (BP) and diabetes are risk factors for dementia/AD; moderate/increasing evidence that midlife high cholesterol and body mass index (BMI) increase the risk of dementia/AD; and moderate/strong evidence that high education and an active lifestyle are protective against dementia/AD. Evidence regarding the effects of smoking and alcohol are still limited due to methodological limitations of previous studies. Interventions on these risk factors have already been conducted regarding cardiovascular disease and type 2 diabetes and similar interventions might also prevent or postpone dementia onset. Retirement is a critical period in adulthood, as loss of work routines may lead to stress and a less healthy lifestyle, i.e. less physical activity, narrow dietary habits, increased alcohol intake. The study population of this study is still at a fragile age (60-74 years) concerning the increase in these risk factors but also at a beneficial age for intervention measures.

Hypertension: High blood pressure in midlife and also later in life and closer to dementia onset is associated with an increased risk of dementia/AD later in life (8). Hypertension has been related to amyloid plaques, neurofibrillary tangles and increased brain atrophy, and may thus be linked to neurodegenerative changes in addition to its known role as a risk factor for cerebrovascular lesions. The Syst-Eur study was the first trial to indicate that active antihypertensive drug treatment may reduce the risk of dementia by 50% (9).

Hypercholesterolemia: High midlife serum total cholesterol (TC) is a risk factor for dementia (8). TC has also been related to AD-type brain changes in autopsy studies (10). Less is known about LDL, HDL and triglycerides. Results from the CAIDE study suggest that, although, high TC in midlife is a risk factor for subsequent AD, decreasing TC later in life may reflect ongoing disease processes and represent a risk marker for dementia (11), i.e. indicating a "reverse causation". Some studies indicate statins as protective against dementia and beneficial even in AD (12) (13).

Obesity and overweight: High BMI in midlife or 9-18 years before the onset of dementia has been associated with an increased risk of AD. Results from the CAIDE study have shown that obesity, high BP and high cholesterol are independent risk factors, roughly doubling the risk of dementia/AD and that multiple risk factors additively increase this risk (14). High BMI has been linked to white matter lesions, greater temporal lobe atrophy and brain atrophy rate.

Diabetes: The relationship of diabetes, insulin resistance, high insulin levels and dementia/AD may reflect direct effects of hyperglycemia and advanced glycation end products on the brain or the effects of diabetes-related co-morbidities (hypertension, dyslipidemia, hyperinsulinemia, etc). The metabolic syndrome, inflammatory markers and increased carotid intima media thickness were recently shown to increase the risk of cognitive impairment in elderly women (15-17).

Dietary factors: It has been shown that moderate intake of unsaturated fats at midlife is protective while saturated fats may increase the risk of AD, especially among ApoE ε4 carriers (18). Studies on AD transgenic mouse models have indicated that essential omega-3 fatty acids protect against neuronal deficits, decrease ß-amyloid levels, and decrease the number of activated microglia in the brain (19). In a recent randomized controlled trial (RCT) patients with very mild AD (MMSE >27) benefitted from omega-3 fatty acids (20). Antioxidants, fish and the 'Mediterranean diet' may be

protective (21). Some studies have linked low levels of vitamin B12 and folate, and high levels of homocysteine to an increased risk of AD.

Active lifestyle: Leisure time physical activity and certain forms of physical activity have been reported to decrease the risk of dementia/AD (22). Results from the CAIDE study showed that regular leisure time physical activity in midlife decreased the risk especially among ApoE ε4 carriers (23). Aerobic exercise improved cardiorespiratory fitness in healthy older people (60-91 years) and led to cognitive improvements (24). Physical activity promotes vascular health and brain plasticity and affects gene transcripts and neurotrophic factors. Social and mental activities may be protective for AD (22). Late-life cognitive training improved targeted cognitive abilities that were sustained over 5 years, but no effects were noted on global cognitive functioning (the ACTIVE study).

Smoking and alcohol consumption: Smoking may result in an increased risk of AD. Heavy/binge drinking may increase dementia risk; a J/U-shaped relation suggests that moderate alcohol intake may be protective. Alcohol may influence several cardiovascular risk factors, and wines contain antioxidants, but also the social and lifestyle related factors associated with certain drinking habits may explain this favorable association (25).

Gene-environment interactions: The effects of life-style related factors (physical inactivity, dietary fat intake, alcohol consumption, smoking) are more pronounced among the ApoE ε4 carriers who developed AD (26). These findings provide an optimistic view for genetically susceptible persons; adopting a healthy lifestyle may lower their risk to the level of an ApoE ε4 non-carrier.

b. Dementia Risk Score

The Dementia Risk Score developed in the CAIDE study is the first tool to estimate the risk of dementia based on midlife risk factors (27, table 1). Similar tools are in use to estimate cardiovascular outcomes and diabetes. The Dementia Risk Score predicted dementia well: AUC 0.77 (0.71-0.83), cut-off >9 points. It was validated in a large diverse population (>10 000 persons) of the Kaiser Permanente study. The Dementia Risk Score is a novel approach and could help to identify the individuals who might benefit from intensive lifestyle consultations and pharmacological interventions.

c. Rationale for a Finnish multi-domain intervention study

The multifactorial and heterogenous character of AD allows multiple prevention approaches but its long preclinical phase makes prevention trials challenging. As many persons are affected, relatively small effects of integrative interventions may have a huge impact on public health. Intervention studies integrating several different approaches have not been done for AD so far, and disappointing results of previous trials with single agents in elderly and already cognitively impaired persons point out some key issues: timing – starting earlier may lead to better effects; target group – a healthy, young population will require very long follow-up times, large sample sizes and considerable financial resources; and outcome measures – cognitive impairment may be a better endpoint than conversion to dementia. Ethical issues also play an important role, as placebo-

controlled trials for high BP and cholesterol are not possible due to their known protective effects regarding cardio- and cerebrovascular disease. These issues and the need for preventive trials were highlighted in the recent Guidelines for AD and other dementias by the European Medicine Agency (EMEA) (http: //www.emea.europa.eu).

The members of this consortium have strong expertise in epidemiological and prevention studies providing rationale for initiating the first multi-domain intervention trial to prevent AD here. The main risk factors for dementia are also those behind cardiovascular diseases and all-cause mortality. Members of our consortium have lead the successful North Karelia Project , the Finnish Diabetes Prevention Study (FDPS) (28), the 6-year exercise intervention trial DNASCO (29) The ongoing exercise and dietary intervention study DR's EXTRA (PI: R. Rauramaa) suggests cardiorespiratory fitness as a component of metabolic syndrome (30) and a predictor of cognitive performance. A systematic review of the efficacy of non-pharmacological therapies in AD concluded that there is increasing evidence of the benefits of physical activity, cognitive stimulation, and multi-component interventions in AD but only a few of the RCT's (8%) were of high quality and further trials with careful methodological considerations are needed (M. Kivipelto part of working group).

Table 1. Dementia Risk Score

**Risk factor**		**Points**	**Score**	**Total score**	**Dementia risk**
**Age**	<47 years<br>47-53 years<br>>53 years	0<br>3<br>4		 **0-5**<br>**6-7**	 **1.0%**<br>**1.9%**
**Gender**	Female<br>Male	0<br>1		**8-9**<br>**10-11**	**4.2%**<br>**7.4%**
**Education**	>10 years<br>7-9 years<br><7 years	0<br>2<br>3		**12-15**	**16.4%**
**Systolic blood pressure**	≤ 140 mmHg<br>>140 mmHg	0<br>2			
**Total cholesterol**	≤ 6.5 mmol/L<br>>6.5 mmol/L	0<br>2			
**Body mass index**	≤ 30 kg/m^2<br>> 30 kg/m^2	0<br>2			
**Physical activity**	Yes<br>No	0<br>1			

3. Aims and hypotheses

The aim of this study is to plan and conduct a 2-year multi-domain intervention study to prevent cognitive impairment and disability. The intervention will focus on common risk factors of cognitive decline for which there is strong evidence. The intervention will consist of four components: i) **Nutrition**, ii) **Exercise**, iii) **Cognitive training** and **social activity**, iv) **Monitoring and management of metabolic and vascular risk factors**.

We hypothesize that this multi-domain intervention will decrease cognitive impairment during two years of follow-up and delay dementia onset (after an extended follow-up up to seven years) among elderly persons with increased dementia risk. The multi-domain intervention is also expected to lower the incidence of depressive symptoms and disability, improve quality of life, and reduce cardiovascular risk factors and related morbidity and mortality. The exploratory hypothesis is that the intervention will also decrease vascular lesions (white matter lesions (WMLs) and small vessel disease) and total brain volume loss on magnetic resonance image (MRI) scans (i.e. the intervention will act both via vascular pathway and protecting against neurodegeneration and/or increasing 'brain reserve').

Significance: The **FINGER** study will be the first RCT to clarify to what extent a carefully designed and monitored multi-domain intervention can delay cognitive impairment and disability among people at an increased dementia risk. The study will also provide information on the mediating pathways. The data will have great scientific value and it is urgently needed for health education and community planning.

4. Methods

Study design: The FINGER study is a multi-center single-blind randomized controlled trial enrolling approximately 1200 independently living persons aged from 5 cities (Helsinki, Kuopio, Oulu, Seinäjoki, Vantaa). Each site will be lead by an experienced sub-group leader and run by a skilled study team.

Recruitment: A random sample of 60-74 year old persons who have previously participated in population-based non-intervention surveys (FINRISK, D2D, Health 2000). The National FINRISK Study is a large population-based survey of cardiovascular risk factors carried out since 1972 every five years using independent, random and representative population samples from different parts of Finland. The National Type 2 Diabetes Prevention Program (FIN-D2D) and the Health 2000 study have used similar methods as FINRISK that comply with international standards. This way of recruitment uses earlier information on participants and, thus, provides unique baseline data for an RCT. Individuals will be screened based on data from these earlier studies using the Dementia Risk Score. Individuals with a score of 8 points or more will be invited to the screening visit for evaluation with the neuropsychological test battery (CERAD).

Inclusion criteria: 1) Modified Dementia Risk Score 8 points or more, and 2) Mild cognitive impairment identified with the Consortium to Establish a Registry for Alzheimer's Disease (CERAD) neuropsychological test battery. For inclusion criteria we will use: 1) Word List Memory task (10 words x3), cut-off: 17 words (based on available data on Finnish population norms (31)),

2) Word List Recall 70%, cut-off: 70%; 3) Mini Mental State Examination, 20-24/30 points. Fulfillment of any one of these CERAD criteria is sufficient for inclusion.

Main **exclusion criteria** at entrance are conditions that inhibit safe engagement in intervention (especially exercise training), malignant diseases, major depression, dementia/substantial cognitive decline (MMSE<20), symptomatic cardiovascular diseases or re-vascularization within 1 year, severe loss of vision, hearing or communicative ability and other conditions preventing from co-operation (32) as judged by the research physician.

Intervention: The study population will be randomized into two groups equal in size to receive an intensive multi-domain intervention or general health advice. The multi-domain intervention will have four main components: i) Nutrition, ii) Exercise, iii) Cognitive & social activity, and iv) Monitoring and management of metabolic and vascular risk factors. The intensive intervention of this study will simultaneously address several common and modifiable risk factors to obtain an optimal prevention effect. The risk and protective factors have been chosen based on the best available knowledge. Persons in the general health advice group will receive advice from the study nurse on a healthy lifestyle and an appropriate level of physical, cognitive, and social activity beneficial for the management of vascular risk factors according to recent recommendations.

INTERVENTION PROTOCOL

The intensive intervention will start with a **Kick-off meeting** during which the background, methods, and aims of each intervention domain will be explained in a 20 minute session by the responsible professional at each site.

1) Diet intervention

Objective

The recommended diet will mainly be based on the Finnish Nutrition Recommendations (33). Recommended diet is also beneficial for individuals with hypertension, dyslipidemia or impaired glucose metabolism and who hence have increased risk for dementia.

In addition to the general recommendations, special emphasis will be on dietary factors which according to earlier findings are associated with dementia risk (21). Factors associated with decreased dementia risk or improved cognitive functions are e.g. n-3 fatty acids, folic acid, vitamin E, and various vitamins from vitamin B group. However, since there is not sufficient evidence for benefits of supplement use, aim will be to achieve adequate nutrient intakes with balanced diet. According to some studies moderate alcohol intake might be protective. Alcohol will not, however, be recommended for abstainers.

Special requirements of the target group will also be taken into consideration while tailoring the dietary intervention. The need for weight loss will always be assessed individually, considering e.g. age, weight history, and general health. Rapid weight loss is not recommended for the elderly and weight maintenance should be achieved with the combination of exercise and balanced diet. In practise intended weight loss might be approximately 5-10 %, which has been shown to efficiently improve metabolic disturbances in overweight (BMI 25-30) and obese (BMI>30) subjects.

Dietary goals in nutrient intake level:

- Protein 10-20 E%
- Total fat 25-35 E%, of which
  - Saturated fatty acids or trans-fatty acids ≤ 10 E%
  - Monounsaturated fatty acids 10-20 E%
  - Polyunsaturated fatty acids 5-10 E%
  - n-3 fatty acids total 2,5-3 g/day, of which alpha-linolenic acid 2 g/day and EPA+DHA (fish fatty acids) 0,5 g/day
- Carbohydrates 45-55 E%, of which refined sugar ≤ 10 E%
- Dietary fibre 25-35 g/day
- Salt (NaCl) ≤ 5 g/day
- Cholesterol ≤ 300 mg/day
- Alcohol ≤ 5 E%

Dietary goals in food intake level:

- Changing from butter and other SFA fats to vegetable fats. Consumption of rapeseed oil and vegetable margarines (≥ 60% fat) ≥ 20 g/day, calculated as oil.
- Consumption of fatty fish (e.g. salmon, Baltic herring) at least 2 portions a week (portion = 125-150 g). For those not consuming fish, fish oil supplements are recommended.
- Consumption of fruit and vegetables according to the recommendation (≥ 500 g/day)
- Choosing whole grain in all cereal products
- Choosing low-fat options in milk products and meat products
- Limiting sucrose intake as such and as ingredient to ≤ 50 g/day
- Consumption of alcoholic beverages max. 2 units/day for men and 1 unit/day for women
- Other goals according to individual needs (e.g. disease, medications, body weight)

If a participant consumes very small amounts of food or otherwise has inadequate diet multivitamin/mineral supplements may be recommended.

Intervention implementation

Dietary intervention will include group sessions and individual counselling. Participants will also have a chance to contact the nutritionist by telephone or e-mail when needed. Additional support will be offered on the internet home page, e.g. tips on food choice, recipes, FAQs and links to useful sites. Study nurse will also contact the intervention group participants regularly by telephone.

- **Individual counselling sessions** (3 face-to-face visits with the study nutritionist during the first year) will include tailoring of diet based on participants' previous everyday diet. All changes are planned together with the participant. Dietary goals will be achieved step by step doing small changes and considering personal features.
- **Group sessions** (7 group sessions during the first year and every 3 months during the second year) will provide more information, motivation and resources aimed at helping to make lifestyle changes. Support of the group will be exploited and sessions will focus on

discussions and practical exercises. Group sessions will be lead be the study nutritionist and spouses are also welcome to join part of the sessions.

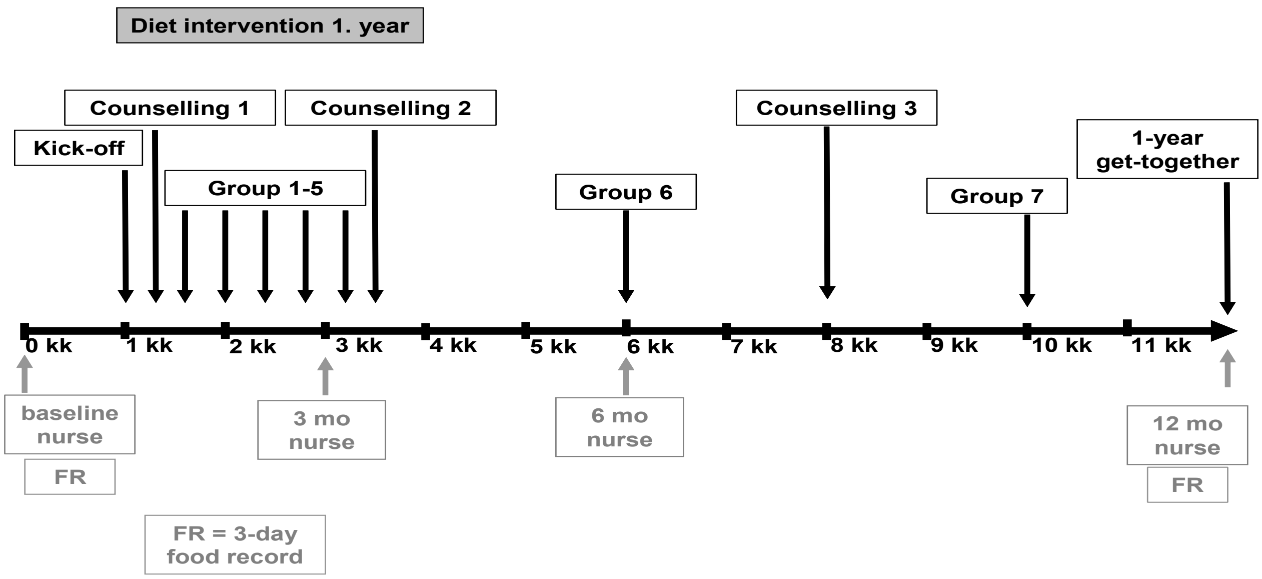

Group sessions will share common themes and same schedule at all study centres ensuring similar intervention content and intensity to all participants. Intervention materials will also be similar for all groups. It is recommended that participants attend the group sessions with the same group throughout the intervention (to facilitate grouping effect), but it is not mandatory. In general participation in group sessions is advisable but not obligatory and subjects will not be excluded even if they do not participate in the group sessions.

Following themes will be discussed in the group meetings

- Fats - quality matters
- Dietary fibre - what it is and why it is important
- Weight management
- Psychology of eating
- Maintaining the changes
- Grocery shopping - does it change and how?
- Package markings and how to read them
- Cooking - how to make it easy and healthy, how to make my favourite dish in a healthier way, how to take advantage of frozen foods and ready meals etc.
- Finding nutrition information on the internet
- Drinks - what should I drink and how much
- Alcohol consumption - pros and cons
- How to eat on special occasion - what should I offer to my grandchildren, what could I eat at a party, how would I manage on holiday

- Tasting and getting to know better options - vegetable margarines, low-fat cheeses etc. everyday foods
- Eating seasonal - what to choose on Christmas, Easter, Midsummer etc.

Control group
Control group participants will receive general information on healthy diet at the baseline visit.

Data collection
Food consumption and nutrient intakes will be assessed by 3-day food records. All subjects record their food consumption at baseline, 12 months and 24 months. Additional information on specific foods (e.g. fish) will be assessed by a frequency questionnaire. Intervention group will monitor their diet and dietary changes during the intervention sessions using specific tools (e.g. tests to assess fat intake or fibre intake).

2) <u>Exercise intervention</u>

Measurements

Exercise will be assessed by 12-month Leisure-Time Physical Activity Questionnaire, DEPAI (DR's EXTRA Physical Activity Interview for the preceding month) at baseline and months 12 and 24, and by a personal notebook.

To define the optimum load for resistance exercise, one repetition maximum (1RM) will be determined at baseline and after 12 and 24 months. Cardiorespiratory fitness will be measured by a maximal symptom-limited exercise test on a cycle ergometer.

Regular health advice group will be given general public health advice on regular physical activity. The subjects in the control group have control visits at 3 months, and 24 months after baseline.

The **exercise training** program is based on the international guidelines (34), **Table 2**. Training intensity will be gradually increased during the first 6 months of the intervention. After baseline strength measurement, the subjects will participate in a supervised, individually prescribed *strength training program*. The loads of the training will be checked at 1, 3, 6, 12, 18 and 24 months after baseline. *Aerobic exercise* will be individually prescribed, moderate-intensity dynamic exercise (producing noticeable increase in heart rate and breathing), occupying large muscle groups. The main exercise modes are walking, skiing, jogging, Nordic walking, biking and swimming. Each participating centre has an obligatory patient insurance.

Table 2. Progression of the resistance and aerobic training program

	0-1 mo	1-3 mo	3-6 mo	6-24 mo
**Resistance Exercise**				
Exercise frequency/wk	1	1	2	2
Duration of warm up, min	5-10	5-10	5-10	5-10
Duration of exercise, min	45 - 60	45 - 60	45 – 60	45 - 60
Number of muscle groups	8-10	8-10	8-10	8-10
Repetitions/ set	10	10	15	15
Load % 1RM	light	40-50	60	60
Number of sets	1	1	1	2
Stretching, min	10	10	10	10
**Aerobic Exercise**				
Exercise frequency/wk	2	3	4	5
Duration of exercise, min	30	30	30	30

3) Cognitive tarining and social activity intervention

Cognitive training will be organized modifying protocols that have been previously shown to be effective in a RCT (35). Training will target memory, reasoning, and speed of processing. The intervention will involve 10 well-structured group sessions lead by a study psychologist. The sessions will last for 60-75 minutes each and be conducted partly in connection with nutritional group sessions. Two booster training sessions will be conducted during the 2nd year. Participation in social and cognitive activity will be monitored (activity diary) during the study. The training protocol will follow that of earlier cognitive training studies (35-37). Memory training will involve teaching mnemonic strategies (organization, visualization, association) for remembering verbal material (e.g. word lists, texts). Reasoning training will involve teaching strategies for finding the pattern in a letter or word series and identifying the next item in the series. Speed of processing training will involve for example visual search and divided attention. Part of the training sessions will focus on applying these strategies to solving everyday problems (e.g. mnemonic strategies to remember a grocery list, reasoning strategies to understand the pattern in a bus schedule).

Social activity will be stimulated throughout the study via the group meetings of the other interventions. Participants will be encouraged to engage in social activities within the intervention group, e.g. Nordic walking together. The local offices of the Alzheimer Society have offered to host meetings to describe their function and activities. Also other organized meetings will be arranged. The amount of participation in social and internet based cognitive activity will be monitored with an activity diary.

The FINGER internet site will be opened with links to memory training programs, sudoku, etc. A chat program will be available on the internet site to promote the possibility of social contacts between the participants. Weekly tips on a healthy lifestyle (recipes, exercise activities) will be provided on the internet site.

4) Intensive monitoring and management of metabolic and vascular risk factors

At baseline all participants will meet the study physician for the evaluation of metabolic and vascular risk factors according to the latest national evidence based guidelines (38-40). Participants of the intensive intervention group whose laboratory test results require attention will be invited to a second physician visit. During this visit they will receive oral and written information on the importance and aims of management of metabolic and vascular risk factors according to current evidence-based guidelines (41-43), be motivated to adhere to the lifestyle changes applicable, and if initiation or adjustment of pharmacologic treatment is necessary they will be strongly recommended to contact their own physician. Participants in the regular health advice group will receive written information regarding their laboratory and other measurements, an information letter on the risks associated with these values, and be strongly recommended to visit their own physician regarding initiation of treatment. The participants will also be strongly motivated to adhere to the pharmacological treatment.

For all participants regular measurements of blood pressure, weight (BMI calculated), and hip-waist ratio will be conducted at month 3, 6, 9, 12, 18 and 24. Total and HDL cholesterol, triglycerides, gamma-glutamyl transferase (GGT), alanine aminotransferase (ALAT), aspartate aminotransferase (ASAT), uric acid, creatinine, C-reactive protein and calcium values will be assessed at baseline, and at months 6, 12, and 24. A 2-hour glucose tolerance test (OGTT) will be performed at baseline and months 12 and 24 including glucose and insulin measurements at 0, 30, 60, and 120 minutes.

Intervention sessions will be frequent during the first 6 months, every 2-3rd month during the next 6 months, and every 3rd month during the 2nd year. Interventions for various components will be synchronized and partly combined.

Study visits: Table 3. The intervention group will meet the study physician at screening, at baseline (if laboratory or other values require attention) and month 6, 12, and 24, and extra visits will be arranged in case of medical problems. They will meet the study nurse at screening, baseline, months 3, 6, 9, 12, 18 and 24 (and during some intervention sessions). The study nurse will also keep telephone contact with the intervention group between the visits (months 1, 5, 15, 20). All participants will have the possibility to call the study nurse if necessary. The regular health advice group will meet the study nurse at screening, baseline, months 6, 12 and 24 and meet the study physician at screening and months 6, 12 and 24.

Blood samples will be taken at baseline, months 6, 12, and 24 to analyze total and HDL cholesterol, TG, ALAT, ASAT, GGT, creatinine, uric acid, CRP, calcium, and the OGTT will be performed (including fasting insulin and glucose). Later we will analyze dietary biomarkers (S-folate, vitamin B-12, homocysteine fatty acid composition), vitamin D, brain derived neurotrophic factor, and zinc. A urine sample will be taken at baseline for future analyses of biomarkers.

Table 3. **Study visits**.

	**Screening**	**Baseline**	**3m**	**6m**	**9m**	**12m**	**18m**	**24m**
**Medical history**	X			X		X		X
**Physical exam**	X			X		X		X

**Weight, BMI, BP**	X	X	X	X	X	X	X	X
**Hip-Waist**		X	X	X	X	X	X	X
**ApoE**		X						
**Lab 1***		X		X		X		X
**Lab 2****		X				X		X
**CERAD********	X			x		x		x
**NTB**		X				X		X
**Zung depression scale**		X		X		X		X
**ADCS-ADL**		X				X		X
**RAND-36, 15D**		X				X		X
**RUD**		X				X		X
**MRI**	X							X
**Dietary FFQ**		X				X		X
**Dietary biomarkers******		X				X		X
**Food records**	X		X	X	X		X	
**Activity diary**		X		X		X		X

*) Lab 1: total cholesterol, HDL, LDL, triglycerides, HbA1c, uric acid, GGT, ALAT, ASAT,
**) Lab 2: OGTT, insulin, CRP
***) Eryhtrocyte fatty acid composition, S-folates, S-B_{12} (active form), homocysteine
****) Only a part of CERAD (MMSE) will be performed at month 6, 12, and 24)

Primary outcome: Cognitive impairment

Cognitive impairment will be measured with the Neuropsychological Test Battery (NTB), a reliable and sensitive measure for mild cognitive changes especially in AD (44) and Trail Making and Stoop Tests to capture executive dysfunction typical in vascular cognitive impairment (45), and incidence of dementia and AD according to standard criteria (DSM-IV and NINCDS-ADRDA). Due to the single-blind nature of the study the NTB will be conducted by a different psychologist than the one leading the cognitive training sessions. During the study participants will be given results of the NTB in relation to population norms. As dementia is one of the outcomes of the study, dementia diagnosis will be made within the scope of the study when necessary for both the intensive intervention and regular health advice groups. The diagnostic procedure will include interview of the proxy using the standard questionnaire (Muistikysely läheiselle).

Secondary outcomes: Disability (ADCS-ADL), Depressive symptoms (Zung scale), Vascular risk factors (BMI, waist- hip ratio, SBP, DBP, PP, serum total cholesterol, HDL, LDL, triglycerides, insulin, CRP, plasma glucose, HbA1c, uric acid, glucose tolerance, creatinine), Dietary biomarkers (Erythrocyte fatty acid composition (a marker of dietary fatty acid intake and fatty acid metabolism), serum folate, S-B_{12} (active form) homocysteine, and Cardiovascular and cerebrovascular morbidity and mortality (registers), Quality of life (measured with RAND-36 and 15D), Utilization of health resources (46). All the scales have been selected according to recent recommendations (e.g. EMEA).

Exploratory outcome: Brain magnetic resonance imaging (MRI) measures will be analyzed for a sub-group of persons (n=100, starting with the Kuopio cohort and, if needed, extending it to the Helsinki cohort). The exploratory hypothesis is that the intervention will also decrease the expression of vascular lesions (white matter lesions and small vessel disease) and total brain and hippocampal volume loss on MRI scans. Structural imaging will be performed using a 1.5T Siemens Avanto (Erlangen, Germany) and the protocol will includeT1, T2, FLAIR and diffusion tensor imaging (DTI) sequences. Images will be read for any abnormalities, and the volumes of hippocampus and entorhinal cortex are measured as described previously (Insausti).

The trial is **single-blind** so that investigators conducting outcome measures are blinded for the randomization group and participants are carefully advised not to discuss the intervention especially during the cognitive testing sessions.

Power calculations: Sample size calculations were based on the expected Neuropsychological Test Battery (NTB) score. Based on previous studies in mild AD (44) we expect a NTB decrease of approx -0.21 z-score with a SD of 0.5 in the placebo arm during 2 years. With 5% significance level and 90% power, the sample size required at the end of trial is approx 500 persons per group to detect a 50% difference in change in NTB score between the two groups. Assuming a drop-out rate of 10% during the trial, starting with 600 per group the power is sufficient. It will also give a possibility to explore possible effect modification by the ApoE ε4 allele and gender differences. During the intervention time (2 years), dementia incidence will still be low in this relatively young population (estimated 10/1000 person-years in the reference group) and no significant difference is expected between the intervention and reference groups (would need >10 000/group). However, an extended follow-up up to 7 years is planned (dementia incidence estimated 20 per 1000) giving 95% power to detect differences expecting that the intervention will lower dementia incidence with 50%.

Statistical methods: Preliminary analyses will involve the univariate examination of the distribution of each of the covariates of interest, in order to identify outliers and assess skewness. When the outcome of interest is the result of a cognitive test we will use linear regression models when test results follow normal distribution. Otherwise distributions will be normalized or variables will be categorized. Several potential confounders will be taken into account. A Repeated Measure, Mixed Model Regression analysis will be applied to compare the trend and the mean change from baseline for NTB and other cognitive tests between the two groups over the 24-month intervention period of the FINGER study. Qualitative parameters will be analyzed using the Cochran-Mantel-Haenszel test. For all other continuous efficacy parameters the change or percentage change from baseline will be analyzed with ANCOVA. The statistical analyses will be conducted using SPSS vr.15 and/or STATA and SAS.

Table 4. Time schedule

	**2009**		**2010**		**2011**		**2012**	
**Planning the intervention, Finalizing ethical approval**								

**Dementia Risk Score, Run-in, Recruitment**								
**Intervention, data collection, analysis**								
**Reporting**								

<u>Time schedule:</u> Table 4. The first half of the year 2009 will be used to refine the knowledge about risk factors, modify the Dementia Risk Score and plan the intervention. Run-in will be started during the second half of 2009 and recruitment will be finished during the first half of 2010. The intervention period will be finished by the first half of 2012. Data will be entered and statistical analyses conducted during the whole study period making prompt reporting possible. If the results indicate the need to increase the power of the trial, it may be decided to extend the intervention for a longer period.

<u>Ethical issues:</u> Each of the earlier studies from which participants for the FINGER study will be recruited (FINRISK, D2D, Health 2000) has been approved by an Ethical Committee. CAIDE: Approved by the Ethics Committee of the Kuopio University Hospital (1st follow-up: 24/97, 07.02.97; 2nd follow-up: 124/2004, 17.08.04). For each of these studies participants have given written informed consent before enrollment in the study. All data files are stored carefully.

The principles of good clinical practice will be applied in the **FINGER** intervention. The lifestyle intervention program will be applied in addition to the latest guidelines of medical therapy. Accordingly, we will refer participants to an appropriate medical care when indicated. Individuals in the regular health advice group will receive a **mini-intervention**, and also meet the study physician and study nurse at screening, baseline (only study nurse), and at 12 and 24 months, and they will have the opportunity for telephone contact with the study nurse throughout the study. Safety issues of the interventions have and will be carefully considered. Prevention of exercise related musculoskeletal and cardiovascular complications during the trial is an essential ethical and scientific issue. The safety of participants will be ensured by individually given instructions regarding proper performance techniques. In case of acute illnesses, deterioration of health status and any other need for additional medical check-up, the participants are advised to consult the study nurse or physician. During the annual control visits special attention will be directed to symptoms and signs indicating need for future medical examination before proceeding with the intervention program. The Finnish National Institute for Health and Welfare has a patient insurance for all participants.

5. Relevance

This project takes an innovative approach to formulating evidence-based preventive measures in cognitive decline and dementia. Primary and secondary outcomes are chosen to ensure transdisciplinarity, as several major disorders in the elderly share the same risk factors and often occur simultaneously. Our team consists of experts in different types of interventions, who are already experienced in working together, and can rapidly act to implement the comprehensive FINGER intervention targeting several risk factors concurrently for an optimal preventive effect. This project will produce both high-quality scientific knowledge and the means to translate it into practice. Such data are urgently needed for health education and community planning.

6. Researchers and research environment

The FINGER consortium is a broad multi-disciplinary team with vast knowledge and experience in the study topic. The consortium is shown in **Table 5**. Docent Miia Kivipelto is the Principal Investigator with overall responsibility for co-ordinating and supervising the sub-studies. A Co-ordination Group from the Institute of Health and Welfare (THL) will co-ordinate the research activities across different centres and take care of data management. The leaders of each sub-group are responsible for the work to be carried out in their groups. Sub-group leaders and key researchers will have yearly meetings to follow the progress of the study, to harmonize activities, and facilitate effective reporting of results. The FINGER study will have a Steering Committee and a Safety Committee.

Table 5. **FINGER consortium and study sites**.

**Name**	**Expertise**	**Role in the project**
**1. National Institute for Health and Welfare**		
**Miia Kivipelto** MD PhD, Associate Prof.	Dementia Epidemiology, Clinical geriatrics, RCTs	Principal investigator; coordinating and supervising sub-studies, supervising PhD students and post docs
**2. Department of Neurology, University of Kuopio and Kuopio University Hospital**		
**Hilkka Soininen**, MD, PhD, Professor	Neurology, Clinical aspects in AD, RCTs, Genetics, Neuroimaging	Sub-group leader, clinical aspects, FINGER Kuopio cohort and MRI sub-study
**Tuomo Hänninen**, PhD, Adjunct Professor	Neuropsychology	Supervising neuropsych. examinations
**3. Department of Chronic Disease Prevention, National Institute for Health and Welfare**		
**Tiina Laatikainen**, MD, PhD, Adjunct Prof.	Epidemiology, Lifestyle interventions	Sub-group leader, FINGER sampling and Vantaa cohort
**Jaana Lindström,** PhD, Senior Researcher	Lifestyle interventions, RCTs	Diet intervention, supervision
**Satu Ahtiluoto**, MD	Clinical geriatrics	Study coordinator, research physician
**4. Department of Public Health, University of Helsinki**		
**Jaakko Tuomilehto**, MD, PhD, Professor	Public health, Cardiovascular, diabetes genetic epidemiology, RCTs and lifestyle interventions	Sub-group leader, Development of risk score, FINGER Seinäjoki cohort, Vascular risk factors intervention, supervision
**5. Department of Geriatrics, University of Kuopio**		
**Raimo Sulkava,** MD, PhD, Professor	Clinical geriatrics	Subgroup leader, GeMS study
**6. Department of Health Sciences/Geriatrics, University of Oulu and Oulu University Hospital**		
**Timo Strandberg,** MD, PhD, Professor	Geriatrics and Medicine, RCTs	Subgroup leader, FINGER Oulu cohort
**7. Kuopio Research Institute of Exercise Medicine**		
**Rainer Rauramaa,** MD, PhD, Professor	Exercmed, Lifestyle interventions RCTs	Subgroup leader, DR's EXTRA, Exercise intervention, supervision

Summary of changes between the original protocol and the final published protocol for the 2-year RCT (*Kivipelto M et al, Alzheimers Dement. 2013;9(6):657-65. doi: 10.1016/j.jalz.2012.09.012,* including the final statistical analysis plan).

Screening was conducted between Sept 7, 2009, and Nov 24, 2011. The 2-year intervention was completed in February 2014. Primary results for the 2-year RCT were published in *Ngandu T et al, the Lancet. 2015;385(9984):2255-2263. doi:10.1016/S0140-6736(15)60461-5*

1. Inclusion criteria were widened to ensure sufficient recruitment in a reasonable time frame. Screening started in September 2009

    a. Age range was changed from 60-74 to 60-77 years (changed in October 2009)

    b. Dementia risk score sufficient to be invited for the study was changed from ≥8 to ≥6 (changed in April 2010)

    c. Screening phase cognitive inclusion criteria were widened in October 2009:

        i. word list learning ≤17 was changed to ≤19

        ii. word list delayed recall ≤70% was changed to ≤75%

        iii. MMSE 20-24 points was changed to 20-26 points

2. Additional study center (Turku) was added in April 2010

3. After the creation of the original study protocol but before the start of first sessions of cognitive intervention, the contents of this intervention were modified

4. Sub-studies/ additional data collection for secondary outcomes were added

5. Time schedule was prolonged

Final protocol for the full FINGER study including extended follow-up visits after the 2-year RCT (ongoing).

FINNISH GERIATRIC INTERVENTION STUDY TO PREVENT COGNITIVE IMPAIRMENT AND DISABILITY

RESEARCH PLAN

1. Abstract
2. Background
3. Aims and hypotheses
4. Methods
5. Relevance
6. Researchers and research environment
7. Financial aspects

1. Abstract

Dementia is a major growing cause of disability in the elderly with the numbers suffering from it proposed to quadruple by the year 2050. This will force enormous pressure on health care systems and society on the whole unless effective means for minimizing dementia incidence are introduced. Evidence from longitudinal population based studies indicates that dementia and its main subgroup Alzheimer's disease share many vascular and life-style related risk factors with cardio- and cerebrovascular diseases. A simple scoring tool, the Dementia Risk Score has recently been developed in the Cardiovascular Risk Factors, Aging and Dementia Study (CAIDE) to predict the risk of late-life dementia based on midlife risk factors. The Finnish Geriatric Intervention Study (FINGER) will plan and conduct a 2-year multi-domain intervention study aiming to lower the risk of cognitive decline in high-risk individuals. The study population of approximately 1200 individuals at an elevated risk of cognitive decline will be screened and recruited from participants of previously randomly sampled study populations. They will be randomized into two groups equal in size to receive either intensive multi-domain intervention or regular health advice. The intervention will consist of four components: nutrition, physical activity, cognitive training and social activity, and intensive monitoring and management of metabolic and vascular risk factors. The primary outcome cognitive decline will be measured using a sensitive neuropsychological test battery. Secondary outcomes to be followed are: disability, depressive symptoms, vascular risk factors and outcomes, quality of life, disability, utilization of health resources and for a subgroup changes noted on brain magnetic resonance imaging. As a two-year period is short for evaluating the slow process of cognitive decline, further follow-ups are planned to fully evaluate the effects of the intervention on the incidence of dementia and the secondary outcomes. We anticipate that this intensive multi-domain intervention on known risk factors will significantly lower the risk of cognitive impairment, postpone the incidence of dementia and have beneficial effects regarding the secondary outcomes. This consortium proposes an innovative approach combining our nations' strong expertise in epidemiological and interventional research to help resolve a major health problem of the coming decades using the scientifically valuable setting of a randomized controlled trial. Our project will provide data urgently needed for the basis of planning health services and education programs related to the prevention of dementia.

2. Background

Alzheimer's disease (AD) is the most common cause of dementia, affecting approximately 24 million people world-wide. This number is estimated to double every 20 years if no effective means of intervention are applied (1). In Finland, approximately 85 000 people have moderate to severe dementia, 35 000 have mild dementia, and 13 000 people develop dementia yearly. In the World Health Report 2003 the disability weight for dementia was higher than for most disorders, only spinal injury and cancer had higher disability weights (2). A 5-year delay in the onset of AD might half the future overall prevalence of AD (3). The key issue is in finding effective preventive or therapeutic means to delay dementia onset.

AD is a multi-factorial syndrome resulting from genetic-environmental interactions. Advanced age, familial aggregation, and the susceptibility gene apolipoprotein E (ApoE) ε4 allele have long been recognized as significant risk factors for AD. These traditional risk factors created a fatalistic view of AD in the past but recent longitudinal studies have indicated several modifiable vascular and life-style related risk factors for AD (4). The identification of modifiable risk factors points to the possibility of delaying or even preventing AD onset. As the process leading to dementia in AD takes decades (5), long-term prospective studies are required to understand the role of the natural course of the disease and its modifiable risk factors. Detailed data on these risk factors is necessary for the development of therapeutic strategies and population education and health programs aiming to prevent or delay AD onset (4). Clinical, genetic and interventional studies are also needed to clarify the pathogenic mechanisms and to translate research findings into practical guidelines to prevent and treat AD efficiently. The ApoE ε4 is relatively common in the Finnish general population (ca 30% and among the demented ca 50%), enabling exploration of effect modification by ApoE ε4 in the current project.

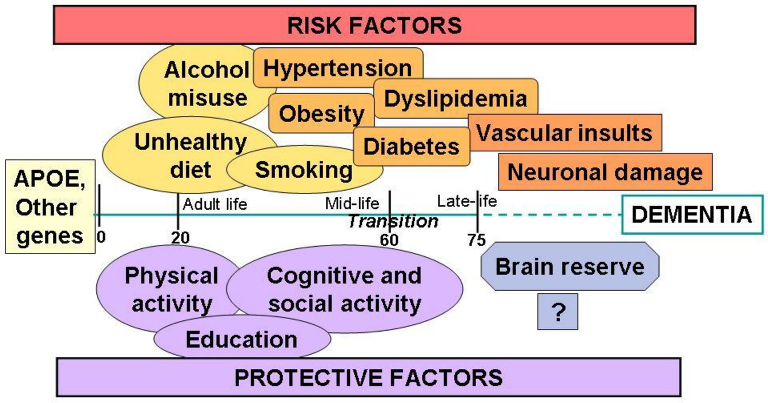

Figure 1. Possible processes and targets of intervention for dementia

a. Modifiable risk factors for dementia and AD

Based on a recent systematic literature review (6) (studies published during 1987-2004) (PI member of the group) and studies published after 2004, there is strong evidence that advanced age and the ApoE ε4 allele are the most important risk factors for dementia/AD; moderate/strong evidence that

high blood pressure (BP) and diabetes are risk factors for dementia/AD; moderate/increasing evidence that midlife high cholesterol and body mass index (BMI) increase the risk of dementia/AD; and moderate/strong evidence that high education and an active lifestyle are protective against dementia/AD. Evidence regarding the effects of smoking and alcohol are still limited due to methodological limitations of previous studies. Interventions on these risk factors have already been conducted regarding cardiovascular disease and type 2 diabetes and similar interventions might also prevent or postpone dementia onset. Retirement is a critical period in adulthood, as loss of work routines may lead to stress and a less healthy lifestyle, i.e. less physical activity, narrow dietary habits, increased alcohol intake. The study population of this study is still at a fragile age (60-77 years) concerning the increase in these risk factors but also at a beneficial age for intervention measures.

Hypertension: High blood pressure in midlife and also later in life and closer to dementia onset is associated with an increased risk of dementia/AD later in life (7). Hypertension has been related to amyloid plaques, neurofibrillary tangles and increased brain atrophy, and may thus be linked to neurodegenerative changes in addition to its known role as a risk factor for cerebrovascular lesions. The Syst-Eur study was the first trial to indicate that active antihypertensive drug treatment may reduce the risk of dementia by 50% (8).

Hypercholesterolemia: High midlife serum total cholesterol (TC) is a risk factor for dementia (7). TC has also been related to AD-type brain changes in autopsy studies (9). Less is known about LDL, HDL and triglycerides. Results from the CAIDE study suggest that, although, high TC in midlife is a risk factor for subsequent AD, decreasing TC later in life may reflect ongoing disease processes and represent a risk marker for dementia (10), i.e. indicating a “reverse causation”. Some studies indicate statins as protective against dementia and beneficial even in AD (11, 12).

Obesity and overweight: High BMI in midlife or 9-18 years before the onset of dementia has been associated with an increased risk of AD. Results from the CAIDE study have shown that obesity, high BP and high cholesterol are independent risk factors, roughly doubling the risk of dementia/AD and that multiple risk factors additively increase this risk (13). High BMI has been linked to white matter lesions, greater temporal lobe atrophy and brain atrophy rate.

Diabetes: The relationship of diabetes, insulin resistance, high insulin levels and dementia/AD may reflect direct effects of hyperglycemia and advanced glycation end products on the brain or the effects of diabetes-related co-morbidities (hypertension, dyslipidemia, hyperinsulinemia, etc). The metabolic syndrome, inflammatory markers and increased carotid intima media thickness were recently shown to increase the risk of cognitive impairment in elderly women (14-16).

Dietary factors: It has been shown that moderate intake of unsaturated fats at midlife is protective while saturated fats may increase the risk of AD, especially among ApoE ε4 carriers (17). Studies on AD transgenic mouse models have indicated that essential omega-3 fatty acids protect against neuronal deficits, decrease ß-amyloid levels, and decrease the number of activated microglia in the brain (18). In a recent randomized controlled trial (RCT) patients with very mild AD (MMSE >27) benefitted from omega-3 fatty acids (19). Antioxidants, fish and the ‘Mediterranean diet’ may be protective (20). Some studies have linked low levels of vitamin B12 and folate, and high levels of homocysteine to an increased risk of AD.

Active lifestyle: Leisure time physical activity and certain forms of physical activity have been reported to decrease the risk of dementia/AD (21). Results from the CAIDE study showed that regular leisure time physical activity in midlife decreased the risk especially among ApoE ε4 carriers (22). Aerobic exercise improved cardiorespiratory fitness in healthy older people (60-91 years) and led to

cognitive improvements (23). Physical activity promotes vascular health and brain plasticity and affects gene transcripts and neurotrophic factors. Social and mental activities may be protective for AD (21). Late-life cognitive training improved targeted cognitive abilities that were sustained over 5 years, but no effects were noted on global cognitive functioning. (24)
Smoking and alcohol consumption: Smoking may result in an increased risk of AD. Heavy/binge drinking may increase dementia risk; a J/U-shaped relation suggests that moderate alcohol intake may be protective. Alcohol may influence several cardiovascular risk factors, and wines contain antioxidants, but also the social and lifestyle related factors associated with certain drinking habits may explain this favorable association. (25)
Gene-environment interactions: The effects of life-style related factors (physical inactivity, dietary fat intake, alcohol consumption, smoking) are more pronounced among the ApoE ε4 carriers who developed AD. (26) These findings provide an optimistic view for genetically susceptible persons; adopting a healthy lifestyle may lower their risk to the level of an ApoE ε4 non-carrier.

b. Dementia Risk Score

The Dementia Risk Score developed in the CAIDE study is the first tool to estimate the risk of dementia based on midlife risk factors (27) (Table 1). Similar tools are in use to estimate cardiovascular outcomes and diabetes. The Dementia Risk Score predicted dementia well: AUC 0.77 (0.71-0.83), cut-off >9 points. It was validated in a large diverse population (>10 000 persons) of the Kaiser Permanente study. The Dementia Risk Score is a novel approach and could help to identify the individuals who might benefit from intensive lifestyle consultations and pharmacological interventions.

c. Rationale for a Finnish multi-domain intervention study

The multifactorial and heterogenous character of AD allows multiple prevention approaches but its long preclinical phase makes prevention trials challenging. As many persons are affected, relatively small effects of integrative interventions may have a huge impact on public health. Intervention studies integrating several different approaches have not been done for AD so far, and disappointing results of previous trials with single agents in elderly and already cognitively impaired persons point out some key issues: timing – starting earlier may lead to better effects; target group – a healthy, young population will require very long follow-up times, large sample sizes and considerable financial resources; and outcome measures – cognitive impairment may be a better endpoint than conversion to dementia. Ethical issues also play an important role, as placebo-controlled trials for high BP and cholesterol are not possible due to their known protective effects regarding cardio- and cerebrovascular disease. These issues and the need for preventive trials were highlighted in the recent Guidelines for AD and other dementias by the European Medicine Agency (EMEA) (http: //www.emea.europa.eu).

The members of this consortium have strong expertise in epidemiological and prevention studies providing rationale for initiating the first multi-domain intervention trial to prevent AD here. The main risk factors for dementia are also those behind cardiovascular diseases and all-cause mortality.

Members of our consortium have lead the successful North Karelia Project , the Finnish Diabetes Prevention Study (FDPS) (28), the 6-year exercise intervention trial DNASCO (29) The ongoing exercise and dietary intervention study DR's EXTRA (PI: R. Rauramaa) suggests cardiorespiratory fitness as a component of metabolic syndrome (30) and a predictor of cognitive performance. A systematic review of the efficacy of non-pharmacological therapies in AD concluded that there is increasing evidence of the benefits of physical activity, cognitive stimulation, and multi-component interventions in AD but only a few of the RCT's (8%) were of high quality and further trials with careful methodological considerations are needed (M. Kivipelto part of working group).

Table 1. Dementia Risk Score

**Risk factor**		**Points**	**Score**	**Total score**	**Dementia risk**
**Age**	<47 years<br>47-53 years<br>>53 years	0<br>3<br>4		**0-5**<br>**6-7**<br>**8-9**<br>**10-11**<br>**12-15**	**1.0%**<br>**1.9%**<br>**4.2%**<br>**7.4%**<br>**16.4%**
**Gender**	Female<br>Male	0<br>1			
**Education**	>10 years<br>7-9 years<br><7 years	0<br>2<br>3			
**Systolic blood pressure**	≤ 140 mmHg<br>>140 mmHg	0<br>2			
**Total cholesterol**	≤ 6.5 mmol/L<br>>6.5 mmol/L	0<br>2			
**Body mass index**	≤ 30 kg/m^2<br>> 30 kg/m^2	0<br>2			
**Physical activity**	Yes<br>No	0<br>1			

3. Aims and hypotheses

The aim of this study is to plan and conduct a 2-year multi-domain intervention study to prevent cognitive impairment and disability. The intervention will focus on common risk factors of cognitive decline for which there is strong evidence. The intervention will consist of four components: i) **Nutrition**, ii) **Exercise**, iii) **Cognitive training** and **social activity**, iv) **Monitoring and management of metabolic and vascular risk factors**.

We hypothesize that this multi-domain intervention will decrease cognitive impairment during two years of follow-up and delay dementia onset (after an extended follow-up up to seven years) among elderly persons with increased dementia risk. The multi-domain intervention is also expected to lower the incidence of depressive symptoms and disability, improve quality of life, and reduce cardiovascular risk factors and related morbidity and mortality. The exploratory hypothesis is that the

intervention will also decrease vascular lesions (white matter lesions (WMLs) and small vessel disease) and total brain volume loss on magnetic resonance image (MRI) scans (i.e. the intervention will act both via vascular pathway and protecting against neurodegeneration and/or increasing 'brain reserve').

Significance: The **FINGER** study will be the first RCT to clarify to what extent a carefully designed and monitored multi-domain intervention can delay cognitive impairment and disability among people at an increased dementia risk. The study will also provide information on the mediating pathways. The data will have great scientific value and it is urgently needed for health education and community planning.

4. Methods

Study design: The FINGER study is a multi-center single-blind randomized controlled trial enrolling approximately 1200 independently living persons aged from 6 cities (Helsinki, Kuopio, Oulu, Seinäjoki, Vantaa, Turku). Each site will be lead by an experienced sub-group leader and run by a skilled study team.

Recruitment: A random sample of 60-77 year old persons who have previously participated in population-based non-intervention surveys (FINRISK, D2D). The National FINRISK Study is a large population-based survey of cardiovascular risk factors carried out since 1972 every five years using independent, random and representative population samples from different parts of Finland. The National Type 2 Diabetes Prevention Program (FIN-D2D) and the Health 2000 study have used similar methods as FINRISK that comply with international standards. This way of recruitment uses earlier information on participants and, thus, provides unique baseline data for an RCT. Individuals will be screened based on data from these earlier studies using the Dementia Risk Score. Individuals with a score of 6 points or more will be invited to the screening visit for evaluation with the neuropsychological test battery (CERAD). The eligible individuals will be asked to confirm their participation by calling the study nurse. If no confirmation is received the study nurse will attempt to contact the individuals by telephone.

Inclusion criteria: 1) Modified Dementia Risk Score 6 points or more, and 2) Mild cognitive impairment identified with the Consortium to Establish a Registry for Alzheimer's Disease (CERAD) neuropsychological test battery. For inclusion criteria we will use: 1) Word List Memory task (10 words x3), cut-off: 19 words (based on available data on Finnish population norms (31)), 2) Word List Recall, cut-off: 75%; 3) Mini Mental State Examination, 20-26/30 points. Fulfillment of any one of these CERAD criteria is sufficient for inclusion.

Main **exclusion criteria** at entrance are conditions that inhibit safe engagement in intervention (especially exercise training), malignant diseases, major depression, dementia/substantial cognitive decline (MMSE<20), symptomatic cardiovascular diseases or re-vascularization within 1 year, severe loss of vision, hearing or communicative ability and other conditions preventing from co-operation (32) as judged by the research physician.

Intervention: The study population will be randomized into two groups equal in size to receive an intensive multi-domain intervention or general health advice. The multi-domain intervention will have four main components: i) Nutrition, ii) Exercise, iii) Cognitive & social activity, and iv) Monitoring and management of metabolic and vascular risk factors. The intensive intervention of this study will simultaneously address several common and modifiable risk factors to obtain an optimal prevention effect. The risk and protective factors have been chosen based on the best available knowledge. Persons in the general health advice group will receive advice from the study nurse on a healthy lifestyle and an appropriate level of physical, cognitive, and social activity beneficial for the management of vascular risk factors according to recent recommendations.

INTERVENTION PROTOCOL

The intensive intervention will start with a **Kick-off meeting** during which the background, methods, and aims of each intervention domain will be explained in a 20 minute session by the responsible professional at each site.

1) Diet intervention

Objective
The recommended diet will mainly be based on the Finnish Nutrition Recommendations (33). The recommended diet is also beneficial for individuals with hypertension, dyslipidemia or impaired glucose metabolism and who hence have increased risk for dementia. In addition to the general recommendations, special emphasis will be on dietary factors which according to earlier findings are associated with dementia risk (20). Factors associated with decreased dementia risk or improved cognitive functions are e.g. n-3 fatty acids, folic acid, vitamin E, and various vitamins from vitamin B group. However, since there is not sufficient evidence for benefits of supplement use, aim will be to achieve adequate nutrient intakes with balanced diet. According to some studies moderate alcohol intake might be protective. Alcohol will not, however, be recommended for abstainers.
Special requirements of the target group will also be taken into consideration while tailoring the dietary intervention. The need for weight loss will always be assessed individually, considering e.g. age, weight history, and general health. Rapid weight loss is not recommended for the elderly and weight maintenance should be achieved with the combination of exercise and balanced diet. In practise intended weight loss might be approximately 5-10 %, which has been shown to efficiently improve metabolic disturbances in overweight (BMI 25-30) and obese (BMI>30) subjects.

Dietary goals in nutrient intake level:

- Protein 10-20 E%
- Total fat 25-35 E%, of which
    - Saturated fatty acids or trans-fatty acids ≤ 10 E%
    - Monounsaturated fatty acids 10-20 E%
    - Polyunsaturated fatty acids 5-10 E%
    - n-3 fatty acids total 2,5-3 g/day, of which alpha-linolenic acid 2 g/day and EPA+DHA (fish fatty acids) 0,5 g/day

- Carbohydrates 45-55 E%, of which refined sugar ≤ 10 E%
- Dietary fibre 25-35 g/day
- Salt (NaCl) ≤ 5 g/day
- Cholesterol ≤ 300 mg/day
- Alcohol ≤ 5 E%

Dietary goals in food intake level:

- Changing from butter and other SFA fats to vegetable fats. Consumption of rapeseed oil and vegetable margarines (≥ 60% fat) ≥ 20 g/day, calculated as oil.
- Consumption of fatty fish (e.g. salmon, Baltic herring) at least 2 portions a week (portion = 125-150 g). For those not consuming fish, fish oil supplements are recommended.
- Consumption of fruit and vegetables according to the recommendation (≥ 500 g/day)
- Choosing whole grain in all cereal products
- Choosing low-fat options in milk products and meat products
- Limiting sucrose intake as such and as ingredient to ≤ 50 g/day
- Consumption of alcoholic beverages max. 2 units/day for men and 1 unit/day for women
- Other goals according to individual needs (e.g. disease, medications, body weight)

If a participant consumes very small amounts of food or otherwise has inadequate diet multivitamin/mineral supplements may be recommended.

Intervention implementation

Dietary intervention will include group sessions and individual counselling. Participants will also have a chance to contact the nutritionist by telephone or e-mail when needed. Additional support will be offered on the internet home page, e.g. tips on food choice, recipes, FAQs and links to useful sites. Study nurse will also contact the intervention group participants regularly by telephone.

- **Individual counselling sessions** (3 face-to-face visits with the study nutritionist during the first year) will include tailoring of diet based on participants' previous everyday diet. All changes are planned together with the participant. Dietary goals will be achieved step by step doing small changes and considering personal features.
- **Group sessions** (7 group sessions during the first year and every 3 months during the second year) will provide more information, motivation and resources aimed at helping to make lifestyle changes. Support of the group will be exploited and sessions will focus on discussions and practical exercises. Group sessions will be lead be the study nutritionist and spouses are also welcome to join part of the sessions.

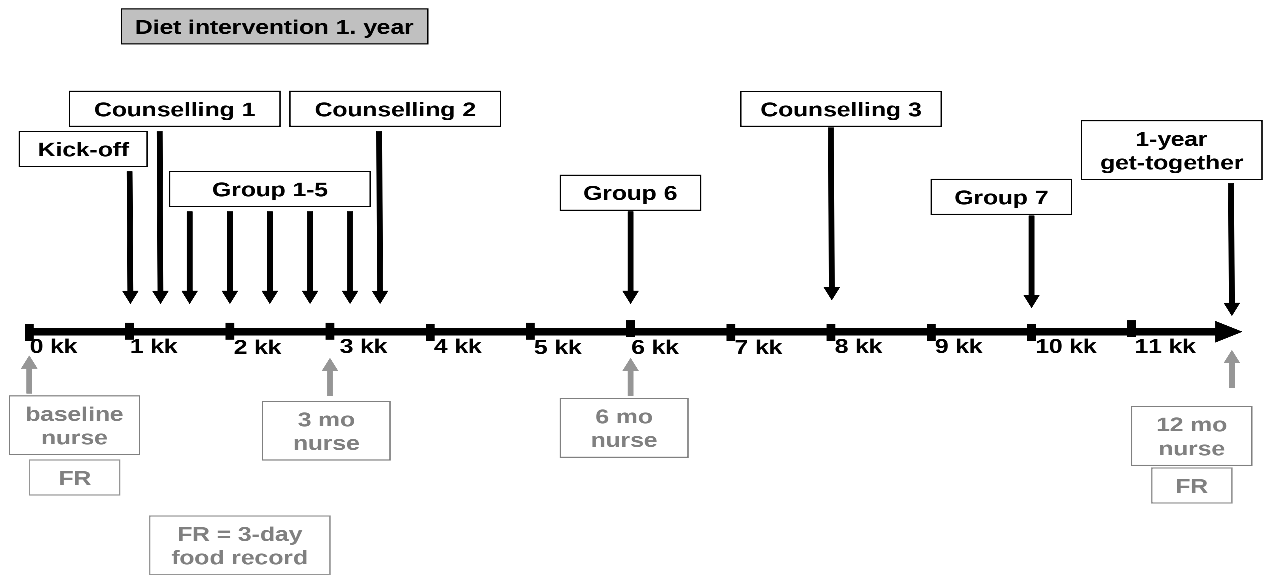

Group sessions will share common themes and same schedule at all study centres ensuring similar intervention content and intensity to all participants. Intervention materials will also be similar for all groups. It is recommended that participants attend the group sessions with the same group throughout the intervention (to facilitate grouping effect), but it is not mandatory. In general participation in group sessions is advisable but not obligatory and subjects will not be excluded even if they do not participate in the group sessions.

Following themes will be discussed in the group meetings

- Fats - quality matters
- Dietary fibre - what it is and why it is important
- Weight management
- Psychology of eating
- Maintaining the changes
- Grocery shopping - does it change and how?
- Package markings and how to read them
- Cooking - how to make it easy and healthy, how to make my favourite dish in a healthier way, how to take advantage of frozen foods and ready meals etc.
- Finding nutrition information on the internet
- Drinks - what should I drink and how much
- Alcohol consumption - pros and cons
- How to eat on special occasion - what should I offer to my grandchildren, what could I eat at a party, how would I manage on holiday
- Tasting and getting to know better options - vegetable margarines, low-fat cheeses etc. everyday foods

- Eating seasonal - what to choose on Christmas, Easter, Midsummer etc.

Control group
Control group participants will receive general information on healthy diet at the baseline visit.

Data collection
Food consumption and nutrient intakes will be assessed by 3-day food records. All subjects record their food consumption at baseline, 12 months and 24 months. Additional information on specific foods (e.g. fish) will be assessed by a frequency questionnaire. Intervention group will monitor their diet and dietary changes during the intervention sessions using specific tools (e.g. tests to assess fat intake or fibre intake). At 7 year follow-up a mini-nutritional assessment will be done to assess risk of malnutrition (33B).

2) <u>Exercise intervention</u>

Measurements

Exercise will be assessed by 12-month Leisure-Time Physical Activity Questionnaire, DEPAI (DR's EXTRA Physical Activity Interview for the preceding month) at baseline and months 12 and 24, and by a personal notebook. Physical status will be assessed at baseline and 24 months by the Short Physical Performance Battery (balance test, timed get-up test, 4m gait velocity) (34), usual gait velocity (10m) measurement, and grip strength measurement, and at 7 years additionally with dual task (counting backwards from 100 while walking) (34B). For the Turku cohort the protocol will also include the Activities-specific Balance confidence (ABC) Scale (35), the perceptual motor speed test (36) and the isometric and dynamic knee extension and flexion measurements.

To define the optimum load for resistance exercise, one repetition maximum (1RM) will be determined at baseline and after 12 and 24 months. Cardiorespiratory fitness will be measured by a maximal symptom-limited exercise test on a cycle ergometer, protocol is shown in Appendix 5.

Regular health advice group will be given general public health advice on regular physical activity. The subjects in the control group have control visits at 3 months, and 24 months after baseline.

The **exercise training** program is based on the international guidelines (37, 38), **Table 2**. Training intensity will be gradually increased during the first 6 months of the intervention. After baseline strength measurement, the subjects will participate in a supervised, individually prescribed *strength training program*. The loads of the training will be checked at 1, 3, 6, 12, 18 and 24 months after baseline. *Aerobic exercise* will be individually prescribed, moderate-intensity dynamic exercise (producing noticeable increase in heart rate and breathing), occupying large muscle groups. The main exercise modes are walking, skiing, jogging, Nordic walking, biking and swimming. Each participating centre has an obligatory patient insurance.

Table 2. Progression of the resistance and aerobic training program

	0-1 mo	1-3 mo	3-6 mo	6-24 mo
**Resistance Exercise**				
Exercise frequency/wk	1-2	1-2	2	2-3
Duration of exercise, min	30- 45	30- 60	45- 60	60
Number of muscle groups	8-10	8-10	8-10	8-10
Repetitions/ set	8-15	10- 20	8- 20	8- 20
Load % 1RM	40-50	60	70	70-80
Number of sets	2	2-3	1-3	2-3
**Aerobic Exercise**				
Exercise frequency/wk	2	2-3	3-4	3-5
Duration of exercise, min	30-45	30-45	30-60	45-60

3) <u>Cognitive training and social activity intervention</u>

Cognitive training will be organized modifying protocols that have been previously shown to be effective in RCTs (39, 40). Training will target episodic memory, mental tempo and working memory. The training will be lead by a psychologist during well-structured group sessions (10 sessions, 60-75 minutes each). Training will include computer-based exercises to enable individually adjusted and progressing difficulty levels. The intervention will consist of two six month independent training periods during which the participants are advised to train on the program for 15 minutes three times a week. Participation in social and cognitive activity will be monitored (activity diary) during the study.

Participants of both groups will be asked to fill out the Memory questionnaire before the intensive intervention group members begin their cognitive training. The participants will also be asked to ask their closest relatives or friends to fill out the Memory questionnaire for the closest relative.

Social activity will be stimulated throughout the study via the group meetings of the other interventions. Participants will be encouraged to engage in social activities within the intervention group, e.g. Nordic walking together. The local offices of the Alzheimer Society have offered to host meetings to describe their function and activities. Also other organized meetings will be arranged. The amount of participation in social and internet based cognitive activity will be monitored with an activity diary.

4) <u>Intensive monitoring and management of metabolic and vascular risk factors</u>

At baseline all participants will meet the study physician for the evaluation of metabolic and vascular risk factors according to the latest national evidence based guidelines (41-43). Participants of the intensive intervention group whose laboratory test results require attention will be invited to a second physician visit. During this visit they will receive oral and written information on the importance and aims of management of metabolic and vascular risk factors according to current evidence-based

guidelines (44-46), be motivated to adhere to the lifestyle changes applicable, and if initiation or adjustment of pharmacologic treatment is necessary they will be strongly recommended to contact their own physician. Participants in the regular health advice group will receive written information regarding their laboratory and other measurements, an information letter on the risks associated with these values, and be strongly recommended to visit their own physician regarding initiation of treatment. The participants will also be strongly motivated to adhere to the pharmacological treatment. This scheme of management will be repeated in connection to laboratory analyses at 6, 12, and 24 months.

Regular measurements of blood pressure, weight (BMI calculated), and hip-waist ratio will be conducted at month 3, 6, 9, 12, 18 and 24 for the intensive intervention group and month 6, 12, and 24 for the regular health advice group. For both groups total and HDL cholesterol, triglycerides, gamma-glutamyl transferase (GGT), alanine aminotransferase (ALAT), aspartate aminotransferase (ASAT), uric acid, creatinine, C-reactive protein, NT-proBNP, and calcium values will be assessed at baseline, and at months 6, 12, and 24. A 2-hour glucose tolerance test (OGTT) will be performed for both groups at baseline and months 12 and 24 including glucose and insulin measurements at 0, 30, and 120 minutes.

Intervention sessions will be frequent during the first 6 months, every 2-3rd month during the next 6 months, and every 3rd month during the 2nd year. Interventions for various components will be synchronized and partly combined.

The participants in the intervention group will be encouraged to achieve and maintain the positive lifestyle changes also after the intervention period by sending them letters that contain information on a healthy lifestyle (nutrition, exercise, cognitive and social activity) according to recent recommendations. One letter per year for five years will be sent. The participants in the intervention group are also given the possibility to freely use the cognitive training program also after the intervention period.

Study visits: Table 3. The intensive intervention group will meet the study physician at screening, at baseline (if laboratory or other values require attention), and months 6, 12, and 24, and extra visits will be arranged in case of medical problems. They will meet the study nurse at screening, baseline, months 3, 6, 9, 12, 18 and 24 (and during some intervention sessions). All participants will have the possibility to call the study nurse if necessary. The regular health advice group will meet the study nurse at screening, baseline, months 6, 12 and 24 and meet the study physician at screening and month 24. Prior to the 12 and 24 month visits participants will be asked to fill in the Health questionnaire and Quality of life questionnaire as applicable.

Follow-up study visits: After the intensive 2 year intervention period, all participants who completed the initial 2-year period (the intervention group and the regular health advice group) and are still alive will be invited to participate in the extended FINGER follow-up visits conducted 5 and 7 years, and 11 and 14 years (+/- 1 year) after baseline. All participants will meet the study nurse and psychologist at the 5-year and 7-year and 11-year and 14-year visits. Participants will be asked to come together with a proxy, and a structured interview (Clinical Dementia Rating scale) will be conducted. All participants suspected to have cognitive impairment based on cognitive test results or concerns

regarding memory expressed by the participant, their proxy or study personnel will be examined by the study physician.

In addition to scheduled visits, the participants will be contacted regularly by telephone to enquire about recent changes in health status, including new diagnoses or medications. Participants may contact the study personnel any time during the follow-up period if necessary.

Drop-out is more likely to occur among participants with cognitive impairment. To minimize drop-out, participants will be offered transportation to the study site if needed, and home visits will be conducted by the study nurse for participants who are unable to come to the study site. Persons who cannot participate in the study site visits or home visits will be contacted by telephone, and a short cognitive test battery (TELE interview)(46 B) will be administered in addition to questions related to health status and reasons for non-participation.

The late follow-up visits (11 and 14 y), postponed to start in 2021 due to global SARS-CoV-2-pandemic (COVID-19), may partly be undertaken during the pandemic. For the safety of the partipants, and for securing participation in the study, a wider range of possibilities for both home visits and remote visits using video calls will be offered in addition to earlier home visits by the nurse and telephone calls. The primary procedure will be visits at the study center, but if the participant prefers, or the current pandemic situation requires, there is a possibility for home visit by the nurse and by the study psychologist (following the regular protocol; including interviews, questionnaires, anthropometrics, and blood samples by the nurse; and neuropsychological test by the psychologist); home visit by the nurse with remote neuropsychological test (following the regular nurse protocol and including a remote contact with psychologist for neuropsychological test, assisted by the nurse doing the dome visit); home visit by the nurse only (regular nurse protocol with brief CERAD test battery and trail making test); or a telephone contact as described in the previous chapter.

Blood samples will be taken at baseline, months 6, 12, 24, 5, 7, 11 years, and 14 years to analyze total and HDL cholesterol, TG, ALAT, ASAT, GGT, creatinine, uric acid, HbA1c, CRP, calcium, fasting glucose (when OGTT is not done), and the OGTT will be performed (including fasting insulin and glucose). Later we will analyze dietary biomarkers (S-folate, vitamin B-12, homocysteine, fatty acid composition), vitamin D, brain derived neurotrophic factor, NT-proBNP, and zinc. A urine sample will be taken at baseline for future analyses of biomarkers.

COVID19 Questionnaire: Between the follow-up data collections 7 and 11 years after the baseline, the global outbreak of SARS-CoV-2- virus causing respiratory illness (the coronavirus disease, COVID-19) emerged, and the pandemic is likely to have serious consequences for health and welfare of senior citizens. Not only are severe and fatal cases of COVID-19 higher in seniors and especially seniors with pre-existing health conditions and multimorbidity (46 C), but also disease management for non-communicable diseases has been reduced due to the pandemic (46 D). Even more importantly, as a non-pharmacological intervention to halt the pandemic, social distancing was introduced in Finland with recommendation for all seniors (+70; the whole FINGER sample in 2020) to voluntarily isolate themselves at home. Thus, there is an urgent need to identify the effects of the COVID-19 outbreak in seniors (70+), especially to identify factors that influence and predict the short- and long-term health outcomes related to coping with lockdown physically and psychosocially. It is also crucial

to estimate the impact of the COVID-19 outbreak and the related control measures for the longer-term effects of the FINGER intervention. Therefore a questionnaire will be added to the FINGER study to evaluate the effects of the pandemic and lockdown, with general aims to i) analyse the impact of COVID-19 outbreak and its management on health, health behavior, function and well-being and ii) estimate the contribution of the COVID-19 and its management on the longer-term effects of FINGER intervention and iii) identify resilience and vulnerability factors for outbreak-related negative outcomes. The questionnaire will be mailed to all participants and they will also be offered a possibility to answer to electronic questionnaire in the internet or to be interviewed by phone.

The questions introduced in the COVID questionnaire for the first time (specific to the pandemic and related behaviour) will be repeated in the follow-up visits at later years to follow up the lifestyle and behavior changes after the pandemic.

Table 3. **Study visits and data collected. Study visits 3m, 9m and 18 m are for the intensive intervention group only**.

	Scr	BL	3m	6m	9m	12m	18m	24m	5y	7y	*CQ*	11y	14y
**Medical history**	X			X		X		X	x	x		x	x
**Physical exam**	X			X		X		X	x	x		x	x
**Weight, BMI, BP**	X	X	X	X	X	X	X	X	x	x		x	x
**Hip-Waist**		X	X	X	X	X	X	X	x	x		x	x
DNA (ApoE allele, epigen.)		X						X		*x*			
**Lab 1**[(1]		X		X		X		X	x	x		x	x
**Lab 2**[(2]		X				X		X		x			
**CERAD**[(3]	X			X		X		X	x	x		x	x
**NTB, Stroop, TMT**		X				X		X	x	x		x	x
**Zung depression scale**		X		X		X		X		x		x	x
**ADCS-ADL**		X				X		X		x		x	x
**RAND-36, 15D**		X				X		X		x		x	x
**Stress, psychol. well-being**										x		x	x
**MRI**	X							X		x		x	x
**Dietary FFQ**		X				X		X		x		x	x
**Dietary biomarkers**[(4]		X		X		X		X		x			
**Food records**		X				X		X		x			
**Mini-Nutritional assessm**										x		x	x
**Activity diary**		X		X		X		X					
**Clinical Dementia Rating**									x	x		x	x
**COVID19 questionnaire**											x	x	x
**Oral health questionnaire**												x	x
**Dental examination**[(5]												x	x
**Qualitative interviews**[6]												x	

1) Lab 1: total cholesterol, HDL, LDL, triglycerides, HbA1c, uric acid, fasting glucose (when OGTT not done), creatinine, NT-proBNP, GGT, ALAT, ASAT,

2) Lab 2: OGTT, insulin, CRP
3) Only a part of CERAD (MMSE) will be performed at month 6
4) Erythrocyte fatty acid composition, S-folate, S-B_{12} (active form), homocysteine
5) For a subsample
6) For a subsample, includes questions related to facilitators and barriers to implementing lifestyle intervention, effects of Covid19 pandemic and factors affecting wellbeing and quality of life

Primary outcome: Cognitive impairment

Cognitive impairment will be measured with the Neuropsychological Test Battery (NTB), a reliable and sensitive measure for mild cognitive changes especially in AD (47) and Trail Making and Stroop Tests to capture executive dysfunction typical in vascular cognitive impairment (48), and incidence of dementia and AD according to standard criteria (DSM-IV and NINCDS-ADRDA). Due to the single-blind nature of the study the NTB will be conducted by a different psychologist than the one leading the cognitive training sessions. During the study participants will be given results of the NTB in relation to population norms. As dementia is one of the outcomes of the study, dementia diagnosis will be made within the scope of the study when necessary for both the intensive intervention and regular health advice groups. The diagnostic procedure will include interview of the proxy and participant using the standard questionnaire (Muistikysely läheiselle and structured Clinical Dementia Rating scale) (49) by a study nurse and a clinical evaluation conducted by the study physician during the medical check-up including the Clinical Dementia Rating scale. Additional assessments for diagnostic purpose, i.e. MRI scans, CSF analysis, PET scans will be carried out when necessary according to standard diagnostic procedure in collaboration with the local memory clinics. We will also ask for written informed consent from the participants to obtain copies of their clinical data from health care centers and hospitals. Final diagnoses will be established by a dementia review board after careful investigation of all available information according to standard criteria. The review board will be blinded to the group status (intervention/ control) of the participant.

Secondary outcomes: Disability (ADCS-ADL and ADL/ IADL 49B, C), Depressive symptoms (Zung scale), Vascular risk factors (BMI, waist- hip ratio, SBP, DBP, PP, serum total cholesterol, HDL, LDL, triglycerides, insulin, CRP, plasma glucose, HbA1c, uric acid, glucose tolerance, creatinine), Dietary biomarkers (Erythrocyte fatty acid composition (a marker of dietary fatty acid intake and fatty acid metabolism), serum folate, S-B_{12} (active form) homocysteine, and Cardiovascular and cerebrovascular morbidity and mortality (registers), Quality of life (measured with RAND-36 and 15D), Utilization of health resources (50). All the scales have been selected according to recent recommendations (e.g. EMEA).

Mild neurocognitive disorder (DSM-5), which represents an at-risk state for dementia will be included as secondary outcome, as well as development of cognitive impairment defined as either Mild or major neurocognitive disorder including dementia.

Exploratory outcomes:

Brain magnetic resonance imaging (MRI) measures will be analyzed for a sub-group of persons (n=100, starting with the Kuopio cohort and, if needed, extending it to the Helsinki, Oulu and Seinäjoki cohorts, and n=100 in Turku cohort). The exploratory hypothesis is that the intervention will also decrease the expression of vascular lesions (white matter lesions and small vessel disease) and

total brain and hippocampal volume loss on MRI scans. Structural imaging will be performed using a 1.5T Siemens Avanto (Erlangen, Germany) and the protocol will include T1, T2, FLAIR and diffusion tensor imaging (DTI) sequences. Images will be read for any abnormalities, and the volumes of hippocampus and entorhinal cortex are measured as described previously (51).

Echocardiography, resting electrocardiogram, ultrasound examination of the right carotid artery, measurement of pulse wave velocity and collection of 24-hour urine for measurements of microalbumin will be done for a sub-group of participants of the Turku cohort (n = approximately 200). The exploratory hypothesis is that the intervention will also decrease progression of intima-media thickness (IMT), carotid artery plaques, stiffness of large arteries, left ventricular hypertrophy and microvascular damage (microalbuminuria), which all are shown to be markers or predictors of cognitive decline. (52-54)

Computerized Cognitive Assessment Battery (CCAB), which is a web-based neuropsychological test similar to CERAD and Trail Making tests, will be conducted for a sub-sample of participants (n approximately 300) at the end of study at the study center and every 12 months at home until end of follow-up period. The hypothesis is that CCAB is a valid instrument in assessment of cognitive function, and that intervention group will have less decrease in CCAB than the control group.

Oral health questionnaire and dental examination will be added to the study protocol in the late follow-up (11 and 14 years). A brief self-reported questionnaire about oral health-related habits and use of services will be sent to all participants and a subsample (n=200, starting from Kuopio cohort and extending to other areas if needed) will be invited to a clinical oral health examination and interview conducted by a dental hygienist and an assistant. Saliva samples will also be collected to assess oral pathogens indicating periodontal diseases. The dental hygienist will conduct clinical oral examinations according to WHO guidelines (55) using mouth mirrors, WHO periodontal probes, and a headlamp with the participant sitting or lying down. The examination includes examination of presence, type, and condition of removable dentures; and an intraoral examination with an examination of the oral mucosa, number and condition of the teeth, measurement of plaques, number of mobile teeth, periodontal condition of teeth, and presence of gingival bleeding. The interview will include questions about use and opinions of oral health services, self-reported oral health including need for care, oral health-related quality of life, and oral health-related behavior.

Qualitative interviews will be added to the study protocol in the late follow-up (11 years). The aim is to gain deeper understanding on facilitators and barriers to lifestyle intervention, effects of Covid19 pandemic and factors contributing to quality of life and wellbeing of the participants. A total of 30-40 persons will be invited to take part in personal and/or group interviews, which will be carried out in person or by using telephone or other remote contact. At first stage, participants belonging to Seinäjoki and Helsinki cohorts will be invited (extended to other areas if needed).We have previously studied health care professionals’ perceptions of FINGER intervention (56), but knowledge on how the participants themselves have perceived the intervention is still lacking.

The trial is **single-blind** so that investigators conducting outcome measures are blinded for the randomization group and participants are carefully advised not to discuss the intervention especially during the cognitive testing sessions.

<u>Power calculations</u>: Sample size calculations were based on the expected Neuropsychological Test Battery (NTB) score. Based on previous studies in mild AD (47) we expect a NTB decrease of approx -0.21 z-score with a SD of 0.5 in the placebo arm during 2 years. With 5% significance level and 90% power, the sample size required at the end of trial is approx 500 persons per group to detect a 50% difference in change in NTB score between the two groups. Assuming a drop-out rate of 10% during the trial, starting with 600 per group the power is sufficient. It will also give a possibility to explore possible effect modification by the ApoE ε4 allele and gender differences. During the intervention time (2 years), dementia incidence will still be low in this relatively young population (estimated 10/1000 person-years in the reference group) and no significant difference is expected between the intervention and reference groups (would need >10 000/group). However, an extended follow-up up to 7 years is planned (dementia incidence estimated 20 per 1000) giving 95% power to detect differences expecting that the intervention will lower dementia incidence with 50%.

<u>Updated power calculation for dementia outcome:</u> Recently, it has been estimated that approximately 30 % of Alzheimer disease would be attributable to modifiable lifestyle-related risk factors (57). In a single-domain prevention trial, a 14 % reduction in incidence of cognitive impairment following intensive blood pressure treatment was observed (58). Based on these recent studies, we expect that the intervention will result in 15-30 % decrease in dementia incidence. A 7 year follow-up is insufficient to demonstrate this. With the original sample size of the FINGER trial (n=1260), approximately 300 dementia cases would be needed to observe HR 0.72 with 80 % power. Based on the age structure of the study population, and the dementia incidence during 2009-2016, this would be achieved in 2024, making the follow-up time approximately 14 years. To observe the HR of 0.85 between groups, even longer follow-up is needed.

<u>Statistical methods</u>: Preliminary analyses will involve the univariate examination of the distribution of each of the covariates of interest, in order to identify outliers and assess skewness. When the outcome of interest is the result of a cognitive test we will use linear regression models when test results follow normal distribution. Otherwise distributions will be normalized or variables will be categorized. Several potential confounders will be taken into account. A Repeated Measure, Mixed Model Regression analysis will be applied to compare the trend and the mean change from baseline for NTB and other cognitive tests between the two groups over the 24-month intervention period of the FINGER study. Dichotomous parameters will be analyzed using the Cochran-Mantel-Haenszel test. For all other continuous efficacy parameters the change or percentage change from baseline will be analyzed with ANCOVA. The statistical analyses will be conducted using SPSS vr.15 and/or STATA and SAS**.**

Table 4. Time schedule

	2009	2010	2011	2012	2013	2014	2015	2016	2017	2018	*2019*	2020	2021	2022	2023
Planning interventions, ethical approval															
Run-in, recruitment															
Intervention, data collection															
Data analysis															
Reporting															
5 and 7 year follow-up data collection															
COVID questionnaire															
11 and 14 year follow-up data collection															

<u>Time schedule:</u> Table 4. The first half of the year 2009 will be used to refine the knowledge about risk factors, modify the Dementia Risk Score and plan the intervention. Run-in will be started during the second half of 2009 and recruitment will be finished during the 2011. The intervention period will be finished by first half of 2014. Data will be entered and statistical analyses conducted during the whole study period making prompt reporting possible. If the results indicate the need to increase the power of the trial, it may be decided to extend the intervention for a longer period. The follow-up visits (5 y and 7 y from baseline) will be conducted starting the first half of 2015 until end of 2018, and the additional follow-up visits (at approximately 11 years and 15 years from baseline) will be conducted starting in 2021 and ending in 2024. Due to COVID19 pandemic, the 11-year data collection may be delayed until the recommendation of isolation is over, but the survey questionnaire has been mailed during the pandemic.

<u>Ethical issues:</u> Each of the earlier studies from which participants for the FINGER study will be recruited (FINRISK, D2D) has been approved by an Ethical Committee. CAIDE: Approved by the Ethics Committee of the Kuopio University Hospital (1st follow-up: 24/97, 07.02.97; 2nd follow-up: 124/2004, 17.08.04). For each of these studies participants have given written informed consent before enrollment in the study. All data files are stored carefully and data protection impact assessments has been completed.

The principles of good clinical practice will be applied in the **FINGER** intervention. The lifestyle intervention program will be applied in addition to the latest guidelines of medical therapy. Accordingly, we will refer participants to an appropriate medical care when indicated. Individuals in the regular health advice group will receive a **mini-intervention**, and also meet the study physician and study nurse at screening, baseline (only study nurse), and at 12 and 24 months, and they will have

the opportunity for telephone contact with the study nurse throughout the study. Safety issues of the interventions have and will be carefully considered. Prevention of exercise related musculoskeletal and cardiovascular complications during the trial is an essential ethical and scientific issue. The safety of participants will be ensured by individually given instructions regarding proper performance techniques. In case of acute illnesses, deterioration of health status and any other need for additional medical check-up, the participants are advised to consult the study nurse or physician. During the annual control visits special attention will be directed to symptoms and signs indicating need for future medical examination before proceeding with the intervention program. The Finnish National Institute for Health and Welfare has a patient insurance for all participants. During the trial some of the participants are expected to develop dementia. In mild state of dementia, the participants are able to give an informed consent. In case of severe dementia, when the participant can no longer give informed consent, a proxy consent will be acquired, and only applicable parts of the data collection are conducted. During the 5-year follow-up visit a separate consent that the data and samples can be collected for the THL biobank and used for biobank research will be asked from the participants. The participants can participate in the follow-up examinations even if they do not give consent to the biobank data collection.

5. Relevance

This project takes an innovative approach to formulating evidence-based preventive measures in cognitive decline and dementia. Primary and secondary outcomes are chosen to ensure transdisciplinarity, as several major disorders in the elderly share the same risk factors and often occur simultaneously. Our team consists of experts in different types of interventions, who are already experienced in working together, and can rapidly act to implement the comprehensive FINGER intervention targeting several risk factors concurrently for an optimal preventive effect. This project will produce both high-quality scientific knowledge and the means to translate it into practice. Such data are urgently needed for health education and community planning.

6. Researchers and research environment

The FINGER consortium is a broad multi-disciplinary team with vast knowledge and experience in the study topic. The consortium is shown in **Table 5**. Professor Miia Kivipelto is the Principal Investigator with overall responsibility for co-ordinating and supervising the sub-studies. A Co-ordination Group from the Institute of Health and Welfare (THL) will co-ordinate the research activities across different centres and take care of data management. The leaders of each sub-group are responsible for the work to be carried out in their groups. Sub-group leaders and key researchers will have yearly meetings to follow the progress of the study, to harmonize activities, and facilitate effective reporting of results. The FINGER study will have a Steering Committee and a Safety Committee. The main collaborator for oral health sub-study is professor Liisa Suominen, DDS, PhD, University of Eastern Finland.

Table 5. FINGER consortium and study sites.

Name	Expertise	Role in the project
**1. Finnish Institute for Health and Welfare**		
**Miia Kivipelto** MD PhD, Prof.	Dementia Epidemiology, Clinical geriatrics, RCTs	Principal investigator; coordinating and supervising sub-studies, supervising PhD students and post docs
**2. Neurology, Institute of Clinical Medicine, University of Eastern Finland and Kuopio University Hospital**		
**Hilkka Soininen**, MD, PhD, Professor	Neurology, Clinical aspects in AD, RCTs, Genetics, Neuroimaging	Sub-group leader, clinical aspects, FINGER Kuopio cohort and MRI sub-study
**Tuomo Hänninen**, PhD, Adjunct Professor	Neuropsychology	Neuropsychological examinations and Cognitive intervention supervision
**Alina Solomon**, MD, PhD, Adjunct Professor	Dementia Epidemiology, RCTs	Scientific coordinator
**3. Department of Public Health and Welfare, Finnish Institute for Health and Welfare**		
**Tiina Laatikainen**, MD, PhD, Prof.	Epidemiology, Lifestyle interventions	Sub-group leader, FINGER sampling and Vantaa cohort
**Markku Peltonen,** PhD, Research Prof.	Epidemiology, Lifestyle interventions, Statistics	Coordination supervision, statistics
**Jaana Lindström,** PhD, Adjunct Professor	Lifestyle interventions, RCTs	Diet intervention, supervision
**Satu Havulinna,** PhD, Senior Researcher	Exercise interventions, fall prevention	Exercise intervention, supervision
**Tiia Ngandu,** MD, PhD, Adjunct Prof.	Clinical geriatrics	Study coordinator**,** Cognitive intervention coordination, research physician
**Jenni Kulmala,** PhD, Associate Professor	Qualitative studies, Epidemiology, gerontology	Coordinator of qualitative interviews
**4. Department of Public Health, University of Helsinki**		
**Jaakko Tuomilehto**, MD, PhD, Professor	Public health, Cardiovascular, diabetes genetic epidemiology, RCTs and lifestyle interventions	Sub-group leader, Development of risk score, FINGER Seinäjoki cohort, Vascular risk factors intervention, supervision
**6. Department of Health Sciences/Geriatrics, University of Oulu and Oulu University Hospital, and University of Helsinki and Helsinki University Hospital/Geriatrics**		
**Timo Strandberg,** MD, PhD, Professor	Geriatrics and Medicine, RCTs	Subgroup leader, FINGER Oulu cohort, Vascular risk factors intervention
**7. Center for Life Course Health Research, University of Oulu and Medical Research Center Oulu, Oulu University Hospital and Oulu City Hospital**		
**Riitta Antikainen**, MD, PhD, Docent	Geriatrics and Medicine, RCTs	Subgroup leader, FINGER Oulu cohort, Vascular risk factors intervention

REFERENCES

1. Ferri, et al. Global prevalence of dementia: a Delphi consensus study. Lancet 2005;366:2112-2117.
2. WorldHealthOrganization. World Health Report 2003 - Shaping the future., 2003.
3. Brookmeyer, et al. Projections of Alzheimer's disease in the United States and the public health impact of delaying disease onset. Am J Public Health 1998;88:1337-1342.
4. Kivipelto, et al. Alzheimer's disease - the ways of prevention. J Nutr Health Aging 2008;12:89S-94S.
5. Braak, et al. Neuropathology of Alzheimer's disease: what is new since A. Alzheimer? Eur Arch Psychiatry Clin Neurosci 1999;249 Suppl 3:14-22.

6. Fratiglioni, et al. Report of the Swedish Council of Technology Assessment in Health Care (SBU) Dementia Risk Factor group, 2006.
7. Kivipelto, et al. Midlife vascular risk factors and Alzheimer's disease in later life: longitudinal, population based study. BMJ 2001;322:1447-1451.
8. Forette, et al. The prevention of dementia with antihypertensive treatment: new evidence from the Systolic Hypertension in Europe (Syst-Eur) study. Arch Intern Med 2002;162:2046-2052.
9. Launer, et al. Cholesterol and neuropathologic markers of AD: a population-based autopsy study. Neurology 2001;57:1447-1452.
10. Solomon, et al. Serum cholesterol changes after midlife and late-life cognition: twenty-one-year follow-up study. Neurology 2007;68:751-756.
11. Solomon, et al. Serum total cholesterol, statins and cognition in non-demented elderly. Neurobiol Aging 2009;30:1006-1009.
12. Sparks, et al. Atorvastatin for the treatment of mild to moderate Alzheimer disease: preliminary results. Arch Neurol 2005;62:753-757.
13. Kivipelto, et al. Obesity and vascular risk factors at midlife and the risk of dementia and Alzheimer disease. Arch Neurol 2005;62:1556-1560.
14. Komulainen, et al. Metabolic syndrome and cognitive function: a population-based follow-up study in elderly women. Dement Geriatr Cogn Disord 2007;23:29-34.
15. Komulainen, et al. Serum high sensitivity C-reactive protein and cognitive function in elderly women. Age Ageing 2007;36:443-448.
16. Komulainen, et al. Carotid intima-media thickness and cognitive function in elderly women: a population-based study. Neuroepidemiology 2007;28:207-213.
17. Eskelinen, et al. Fat intake at midlife and cognitive impairment later in life: a population-based CAIDE study. Int J Geriatr Psychiatry 2008;23:741-747.
18. Oksman, et al. Impact of different saturated fatty acid, polyunsaturated fatty acid and cholesterol containing diets on beta-amyloid accumulation in APP/PS1 transgenic mice. Neurobiol Dis 2006;23:563-572.
19. Freund-Levi, et al. Omega-3 fatty acid treatment in 174 patients with mild to moderate Alzheimer disease: OmegAD study: a randomized double-blind trial. Arch Neurol 2006;63:1402-1408.
20. Luchsinger, et al. Dietary factors and Alzheimer's disease. Lancet Neurol 2004;3:579-587.
21. Fratiglioni, et al. An active and socially integrated lifestyle in late life might protect against dementia. Lancet Neurol 2004;3:343-353.
22. Rovio, et al. Leisure-time physical activity at midlife and the risk of dementia and Alzheimer's disease. Lancet Neurol 2005;4:705-711.
23. Angevaren, et al. Physical activity and enhanced fitness to improve cognitive function in older people without known cognitive impairment. Cochrane Database Syst Rev 2008:CD005381.
24. Willis, et al. Long-term effects of cognitive training on everyday functional outcomes in older adults. JAMA 2006;296:2805-2814.
25. Anttila, et al. Alcohol drinking in middle age and subsequent risk of mild cognitive impairment and dementia in old age: a prospective population based study. BMJ 2004;329:539.
26. Kivipelto, et al. Apolipoprotein E epsilon4 magnifies lifestyle risks for dementia: a population-based study. J Cell Mol Med 2008;12:2762-2771.
27. Kivipelto, et al. Risk score for the prediction of dementia risk in 20 years among middle aged people: a longitudinal, population-based study. Lancet Neurol 2006;5:735-741.
28. Tuomilehto, et al. Prevention of type 2 diabetes mellitus by changes in lifestyle among subjects with impaired glucose tolerance. N Engl J Med 2001;344:1343-1350.
29. Rauramaa, et al. Effects of aerobic physical exercise on inflammation and atherosclerosis in men: the DNASCO Study: a six-year randomized, controlled trial. Ann Intern Med 2004;140:1007-1014.
30. Hassinen, et al. Cardiorespiratory fitness as a feature of metabolic syndrome in older men and women: the Dose-Responses to Exercise Training study (DR's EXTRA). Diabetes Care 2008;31:1242-1247.
31. Pulliainen V. Muistihäiriöiden seulonta - suomalaiset normit CERAD-tehtäväsarjalle. Suomen Lääkärilehti 2007;62:1235-1241.

32.Bjarnason-Wehrens, et al. Recommendations for resistance exercise in cardiac rehabilitation. Recommendations of the German Federation for Cardiovascular Prevention and Rehabilitation. Eur J Cardiovasc Prev Rehabil 2004;11:352-361.
33.NationalNutritionCouncil. Finnish Nutrition Recommendations - Diet and physical activity in balance, 2005.
33B. Rubenstein, et al. Screening for undernutrition in geriatric practice: developing the short-form mini-nutritional assessment (MNA-SF). J Gerontol A Biol Sci Med Sci. 2001 Jun;56(6):M366-72.
34.Guralnik, et al. A short physical performance battery assessing lower extremity function: association with self-reported disability and prediction of mortality and nursing home admission. J Gerontol 1994;49:M85-94.
34B.Montero-Odasso M. Quantitative gait analysis under dual-task in older people with mild cognitive impairment: a reliability study. J Neuroeng Rehabil. 2009 Sep 21;6:35. doi: 10.1186/1743-0003-6-35.
35.Powell, et al. The Activities-specific Balance Confidence (ABC) Scale. J Gerontol A Biol Sci Med Sci 1995;50A:M28-34.
36.Health 2000 Survey Methodology Report. Helsinki: KTL - National Public Health Institute, 2008.
37.Nelson, et al. Physical activity and public health in older adults: recommendation from the American College of Sports Medicine and the American Heart Association. Circulation 2007;116:1094-1105.
38.Physical Activity Guidelines Advisory Committee report, 2008. To the Secretary of Health and Human Services. Part A: executive summary. Nutr Rev 2009;67:114-120.
39.Dahlin, et al. Transfer of learning after updating training mediated by the striatum. Science 2008;320:1510-1512.
40.Dahlin, et al. Plasticity of executive functioning in young and older adults: immediate training gains, transfer, and long-term maintenance. Psychol Aging 2008;23:720-730.
41.Suomalaisen Lääkäriseuran Duodecimin, Diabetesliiton Lääkärineuvoston ja Suomen sisätautilääkärien yhdistyksen asettama työryhmä. Diabeteksen Käypä hoito -suositus. Duodecim 2009;123:1489-1520. Duodecim Artikkelin tunnus: hoi50056 (050.056). http://www.kaypahoito.fi
42.Suomalaisen Lääkäriseuran Duodecimin ja Suomen Verenpaineyhdistys ry:n asettama työryhmä. Kohonnut verenpaine Käypä Hoito -suositus. 2009. Duodecim Artikkelin tunnut: hoi04010 (004.010). http://www.kaypahoito.fi
43.Suomalaisen Lääkäriseuran Duodecimin ja Suomen Sisätautilääkärien Yhdistys ry:n asettama työryhmä. Dyslipidemiat Käypä hoito -suositus. 2009. Duodecim Artikkelin tunnus: hoi50025 (050.025). http://www.kaypahoito.fi
44.Suomalainen Lääkäriseura Duodecim. Käyvän hoidon potilasversio: Kolesterolin ja veren muiden rasvojen häiriöt. 2009. http://www.kaypahoito.fi
45.Suomalainen Lääkäriseura Duodecim. Käyvän hoidon potilasversio: Verenpaine. Käyvän hoidon potilasversiot. 2009. http://www.kaypahoito.fi
46.Suomalainen Lääkäriseura Duodecim. Käyvän hoidon potilasversio: Lihavuus. Käyvän hoidon potilasversiot. 2009. http://www.kaypahoito.fi
46 B.Järvenpää, et al. Characteristics of two telephone screens for cognitive impairment. Dement Geriatr Cogn Disord 2002;13:149-155.
46 C. Onder G, Rezza G, Brusaferro S. Case-Fatality Rate and Characteristics of Patients Dying in Relation to COVID-19 in Italy.JAMA. 2020 Mar 23. [Epub ahead of print]
46 D.Willan J, King AJ, Jeffery K, Bienz N. Challenges for NHS hospitals during covid-19 epidemic.
BMJ. 2020 Mar 20;368:m1117.
47.Harrison, et al. Arch Neurol 2007; 64:1323-1329.
48. Hashinski, et al. Stroke 2006;37: 2220-2241.
49. Aisen, et al. Report of the task force on designing clinical trials in early (predementia) AD. Neurology 2011;76:280.
49 B. Katz et al. Studies of illness in the aged. The index of ADL: A standardized measure of biological and psychosocial function. JAMA. 1963;185:914-919.
49 C.Lawton et al. Assessment of older people: Self-maintaining and instrumental activities of daily living. Gerontologist. 1969;9(3):179-186.
50.Terveyspalvelujen käyttö ja sen väestöryhmittäiset erot. Terveys 2000 –tutkimus, KTL B10/2006

51.Insausti, et al. MR volumetric analysis of the human entorhinal, perirhinal, and temporopolar cortices. Am J Neuroradiol 1998;19:659-671.
52.Triantafyllidi, et al. Cognitive impairment is related to increased arterial stiffness and microvascular damage in patients with never-treated essential hypertension. Am J Hypertens 2009;22:525-530.
53.Scuteri, et al. Left ventricular mass increae is associated with cognitive decline and dementia in the elderly independdetly of blood pressure. Eur Heart J 2009;30:1525-1529.
54.Bruce, et al. Predictors of cognitive decline in older individuals with diabetes. Diabetes Care 2008;31:2103-2107.
55. WHO. Oral Health Surveys. Basics Methods. 4th ed. Geneva: WHO; 1997.
56. Kulmala, et al. Facilitators and barriers to implementing lifestyle intervention program to prevent cognitive decline (Eur J Public Health, in press)
57.Norton, et al. Potential for primary prevention of Alzheimer's disease: an analysis of population-based data. Lancet Neurol. 2014;13:788-94.
58. SPRINT MIND Investigators for the SPRINT Research Group, Williamson, et al. Effect of Intensive vs Standard Blood Pressure Control on Probable Dementia: A Randomized Clinical Trial.JAMA. 2019;321:553-561.
