# Supplementary material for: Brain patterns and risk factors in the FINGER RCT multimodal lifestyle intervention
Source: J Prev Alzheimers Dis. 2025 Sep 24;12(10):100390. doi: 10.1016/j.tjpad.2025.100390 (PMC12627893; doi:10.1016/j.tjpad.2025.100390)
Supplement: Supplementary file 4 [file mmc4.docx]

**Table of Contents**

[**Supplementary Figure 1.** 2](#_Toc206418106)

[**Supplementary Figure 2.** 3](#_Toc206418107)

[**Supplementary Table 1.** 4](#_Toc206418108)

[**Supplementary Table 2.** 6](#_Toc206418109)

[**Supplementary Table 3.** 7](#_Toc206418110)

[**Supplementary Table 4.** 8](#_Toc206418111)

## **Supplementary Figure 1.**

**(A)** Scatter plot of the selected principal components (Component 1 and Component 3) from the random forest output, illustrating cortical thickness and volume heterogeneity in a 2D multidimensional scaling (MDS) visualization of the similarity matrix. Different colours represent distinct clusters identified in the data. These components were chosen after visually inspecting all combinations of Components 1, 2, and 3, following the random forest protocol. The selection aimed to maximize the visualization of heterogeneity while minimizing the influence of other variability sources.

**(B)** Hierarchical clustering tree result. The horizontal axis represents the distance in random forest similarity between the subjects, while the vertical axis shows the hierarchical grouping based on similarities. Colours represent different subtypes as indicated in the legend.


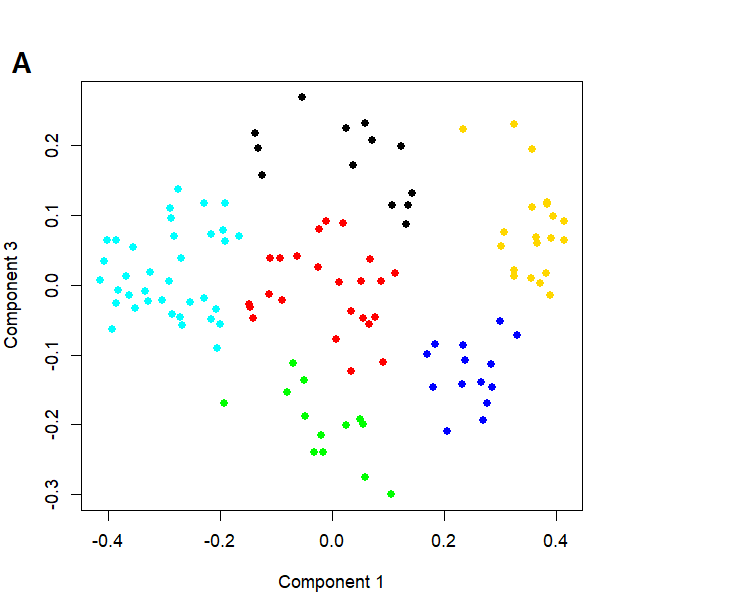


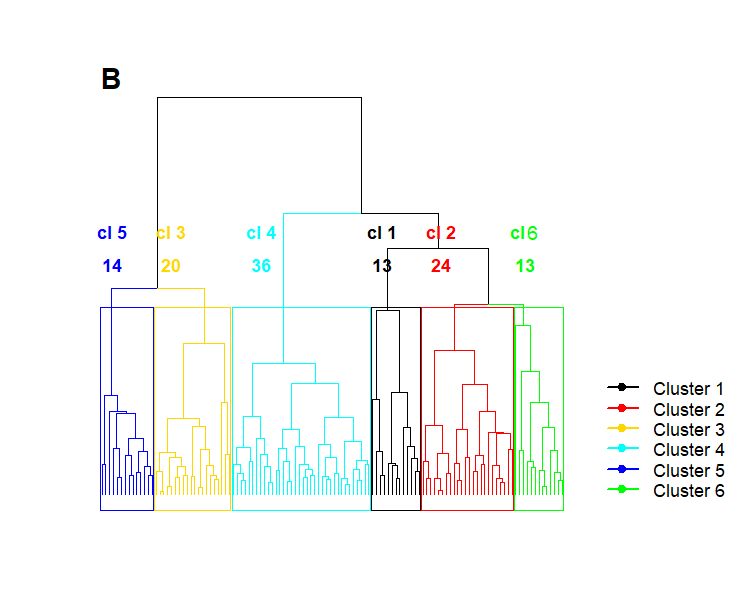


## **Supplementary Figure 2.**

The six identified patterns of grey matter cortical thickness (left) and subcortical volume (right) with respect to the overall mean. Cold colours (blue/light blue) indicate greater thickness/volume, whereas hot colours (red/yellow) indicate greater atrophy. The colour bar on the bottom-right corner denotes Cohen’s d effect sizes. Grey colour indicates non-significant difference from the reference. Right and left hemispheres are averaged. For visualization purposes, we show left lateral and medial view for cortical regions and left coronal view for subcortical regions.


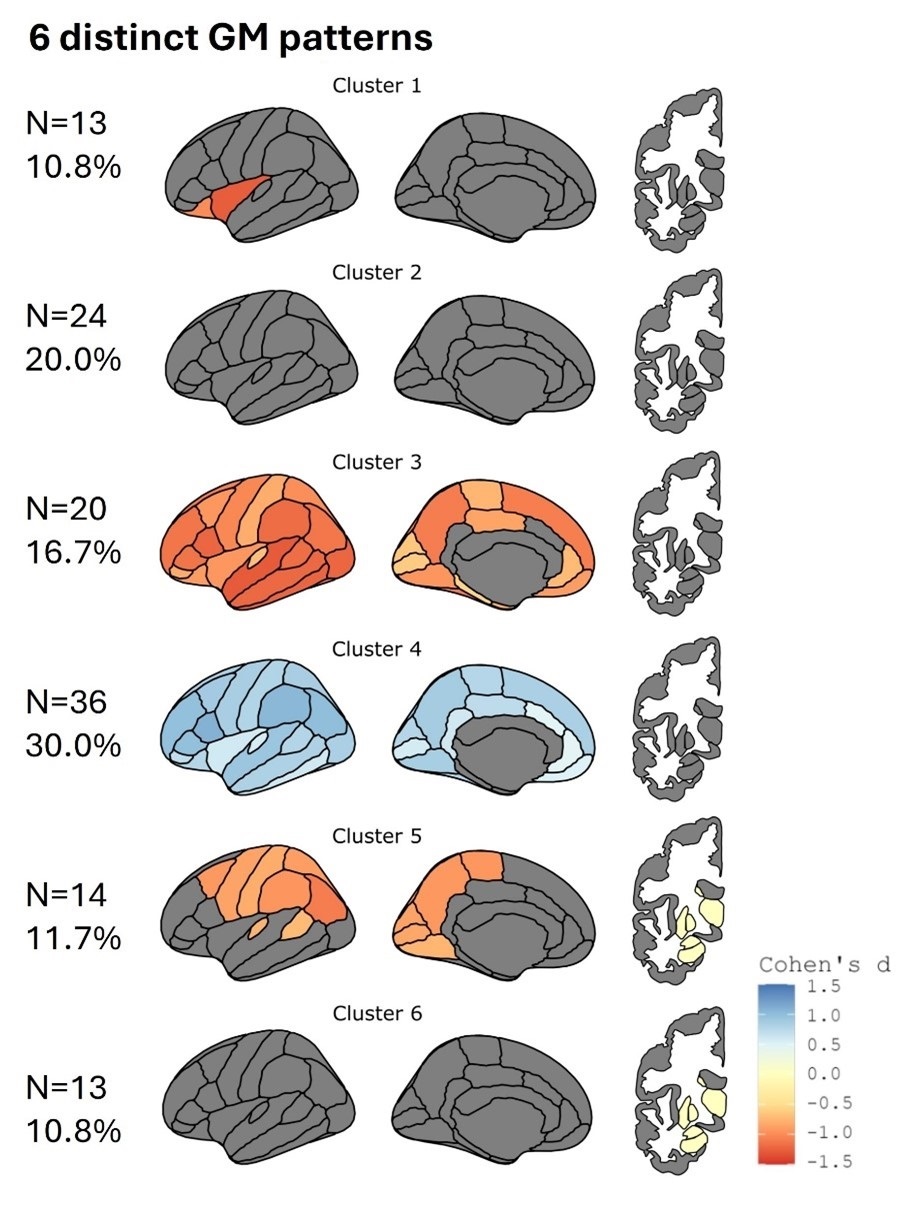


## **Supplementary Table 1.**

Sociodemographic, vascular, lifestyle, and medical characteristics across clusters at baseline.

|  | 1 (N=13) | 2 (N=24) | 3 (N=20) | 4 (N=36) | 5 (N=14) | 6 (N=13) | Tot (N=120) | p |
| --- | --- | --- | --- | --- | --- | --- | --- | --- |
| Sociodemographic characteristics |  |  |  |  |  |  |  |  |
| Study group |  |  |  |  |  |  |  | 0.668 |
| -  Control | 7 (53.8%) | 8 (33.3%) | 7 (35.0%) | 21 (58.3%) | 8 (57.1%) | 8 (61.5%) | 59 (49.2%) |  |
| -  Intervention | 6 (46.2%) | 16 (66.7%) | 13 (65.0%) | 15 (41.7%) | 6 (42.9%) | 5 (38.5%) | 61 (50.8%) |  |
| Age |  |  |  |  |  |  |  | 0.575 |
| -  Mean | 69.7 | 69.5 | 70.8 | 68.7 | 71.1 | 72.2 | 70.0 |  |
| -  SD | 4.9 | 4.5 | 5.6 | 5.0 | 3.6 | 2.5 | 4.7 |  |
| Sex |  |  |  |  |  |  |  | 0.434 |
| -  Men | 12 (92.3%) | 10 (41.7%) | 10 (50.0%) | 16 (44.4%) | 6 (42.9%) | 7 (53.8%) | 61 (50.8%) |  |
| -  Women | 1 (7.7%) | 14 (58.3%) | 10 (50.0%) | 20 (55.6%) | 8 (57.1%) | 6 (46.2%) | 59 (49.2%) |  |
| Years of education |  |  |  |  |  |  |  | 0.750 |
| -  Mean | 10.2 | 9.7 | 9.3 | 9.4 | 9.0 | 8.0 | 9.3 |  |
| -  SD | 3.6 | 3.0 | 2.3 | 2.2 | 2.5 | 1.7 | 2.6 |  |
| Married or cohabiting |  |  |  |  |  |  |  | 0.881 |
| -  N-Miss | 0 | 0 | 1 | 0 | 0 | 0 | 1 |  |
| -  No | 1 (7.7%) | 4 (16.7%) | 5 (26.3%) | 7 (19.4%) | 3 (21.4%) | 4 (30.8%) | 24 (20.2%) |  |
| -  Yes | 12 (92.3%) | 20 (83.3%) | 14 (73.7%) | 29 (80.6%) | 11 (78.6%) | 9 (69.2%) | 95 (79.8%) |  |
| Vascular factors |  |  |  |  |  |  |  |  |
| Systolic blood pressure |  |  |  |  |  |  |  | 0.945 |
| -  Mean | 139.4 | 135.0 | 139.1 | 138.0 | 138.3 | 141.0 | 138.1 |  |
| -  SD | 16.4 | 15.9 | 15.3 | 13.6 | 8.7 | 18.9 | 14.6 |  |
| Diastolic blood pressure |  |  |  |  |  |  |  | 0.750 |
| -  Mean | 81.4 | 77.0 | 79.2 | 80.0 | 77.4 | 76.0 | 78.7 |  |
| -  SD | 7.8 | 7.7 | 10.8 | 8.2 | 6.9 | 10.3 | 8.6 |  |
| Cholesterol |  |  |  |  |  |  |  | 0.511 |
| -  Mean | 4.8 | 4.8 | 4.9 | 5.3 | 4.5 | 5.1 | 5.0 |  |
| -  SD | 1.0 | 0.8 | 1.0 | 1.1 | 0.8 | 0.6 | 0.9 |  |
| Glucose after 2 hours |  |  |  |  |  |  |  | 0.434 |
| -  Mean | 8.2 | 6.5 | 6.9 | 6.1 | 7.1 | 7.1 | 6.7 |  |
| -  SD | 2.1 | 1.9 | 1.8 | 1.5 | 1.2 | 2.5 | 1.9 |  |
| BMI |  |  |  |  |  |  |  | 0.511 |
| -  Mean | 28.2 | 27.2 | 26.2 | 27.3 | 28.8 | 29.0 | 27.6 |  |
| -  SD | 2.5 | 4.0 | 3.1 | 3.2 | 3.8 | 3.8 | 3.5 |  |
| Lifestyle |  |  |  |  |  |  |  |  |
| Physical activity min 2/week |  |  |  |  |  |  |  | 0.945 |
| -  N-Miss | 1 | 0 | 0 | 0 | 2 | 1 | 4 |  |
| -  No | 2 (16.7%) | 7 (29.2%) | 5 (25.0%) | 8 (22.2%) | 4 (33.3%) | 3 (25.0%) | 29 (25.0%) |  |
| -  Yes | 10 (83.3%) | 17 (70.8%) | 15 (75.0%) | 28 (77.8%) | 8 (66.7%) | 9 (75.0%) | 87 (75.0%) |  |
| Smoking |  |  |  |  |  |  |  | 0.881 |
| -  N-Miss | 0 | 4 | 1 | 1 | 0 | 2 | 8 |  |
| -  No | 13 (100.0%) | 19 (95.0%) | 19 (100.0%) | 33 (94.3%) | 14 (100.0%) | 10 (90.9%) | 108 (96.4%) |  |
| -  Yes | 0 (0.0%) | 1 (5.0%) | 0 (0.0%) | 2 (5.7%) | 0 (0.0%) | 1 (9.1%) | 4 (3.6%) |  |
| Alcohol min 1/week |  |  |  |  |  |  |  | 0.750 |
| -  N-Miss | 0 | 0 | 1 | 0 | 0 | 0 | 1 |  |
| -  No | 5 (38.5%) | 9 (37.5%) | 8 (42.1%) | 14 (38.9%) | 8 (57.1%) | 9 (69.2%) | 53 (44.5%) |  |
| -  Yes | 8 (61.5%) | 15 (62.5%) | 11 (57.9%) | 22 (61.1%) | 6 (42.9%) | 4 (30.8%) | 66 (55.5%) |  |
| Fish min 2/week |  |  |  |  |  |  |  | 0.465 |
| -  N-Miss | 0 | 0 | 0 | 1 | 1 | 0 | 2 |  |
| -  No | 8 (61.5%) | 11 (45.8%) | 8 (40.0%) | 17 (48.6%) | 1 (7.7%) | 4 (30.8%) | 49 (41.5%) |  |
| -  Yes | 5 (38.5%) | 13 (54.2%) | 12 (60.0%) | 18 (51.4%) | 12 (92.3%) | 9 (69.2%) | 69 (58.5%) |  |
| Vegetables min 2/week |  |  |  |  |  |  |  | 0.750 |
| -  No | 5 (38.5%) | 4 (16.7%) | 8 (40.0%) | 12 (33.3%) | 5 (35.7%) | 6 (46.2%) | 40 (33.3%) |  |
| -  Yes | 8 (61.5%) | 20 (83.3%) | 12 (60.0%) | 24 (66.7%) | 9 (64.3%) | 7 (53.8%) | 80 (66.7%) |  |
| Self-reported medical conditions |  |  |  |  |  |  |  |  |
| Hypertension |  |  |  |  |  |  |  | 0.511 |
| -  N-Miss | 0 | 2 | 2 | 0 | 0 | 0 | 4 |  |
| -  No | 6 (46.2%) | 7 (31.8%) | 2 (11.1%) | 15 (41.7%) | 6 (42.9%) | 7 (53.8%) | 43 (37.1%) |  |
| -  Yes | 7 (53.8%) | 15 (68.2%) | 16 (88.9%) | 21 (58.3%) | 8 (57.1%) | 6 (46.2%) | 73 (62.9%) |  |
| Hypercholesterolemia |  |  |  |  |  |  |  | 0.945 |
| -  N-Miss | 0 | 2 | 2 | 0 | 0 | 0 | 4 |  |
| -  No | 3 (23.1%) | 8 (36.4%) | 4 (22.2%) | 9 (25.0%) | 3 (21.4%) | 4 (30.8%) | 31 (26.7%) |  |
| -  Yes | 10 (76.9%) | 14 (63.6%) | 14 (77.8%) | 27 (75.0%) | 11 (78.6%) | 9 (69.2%) | 85 (73.3%) |  |
| Diabetes (history) |  |  |  |  |  |  |  | 0.750 |
| -  N-Miss | 0 | 2 | 2 | 0 | 0 | 0 | 4 |  |
| -  No | 2 (15.4%) | 6 (27.3%) | 4 (22.2%) | 3 (8.3%) | 4 (28.6%) | 4 (30.8%) | 23 (19.8%) |  |
| -  Yes | 11 (84.6%) | 16 (72.7%) | 14 (77.8%) | 33 (91.7%) | 10 (71.4%) | 9 (69.2%) | 93 (80.2%) |  |
| Diabetes (current) |  |  |  |  |  |  |  | 0.434 |
| -  N-Miss | 0 | 2 | 2 | 0 | 0 | 0 | 4 |  |
| -  No | 11 (84.6%) | 16 (72.7%) | 14 (77.8%) | 33 (91.7%) | 10 (71.4%) | 9 (69.2%) | 93 (80.2%) |  |
| -  Prediabetes | 0 (0.0%) | 5 (22.7%) | 1 (5.6%) | 2 (5.6%) | 0 (0.0%) | 1 (7.7%) | 9 (7.8%) |  |
| -  Diabetes | 2 (15.4%) | 1 (4.5%) | 3 (16.7%) | 1 (2.8%) | 4 (28.6%) | 3 (23.1%) | 14 (12.1%) |  |
| Heart failure last year |  |  |  |  |  |  |  | 0.750 |
| -  N-Miss | 0 | 2 | 2 | 0 | 0 | 0 | 4 |  |
| -  No | 13 (100.0%) | 22 (100.0%) | 16 (88.9%) | 35 (97.2%) | 13 (92.9%) | 12 (92.3%) | 111 (95.7%) |  |
| -  Yes | 0 (0.0%) | 0 (0.0%) | 2 (11.1%) | 1 (2.8%) | 1 (7.1%) | 1 (7.7%) | 5 (4.3%) |  |
| Cerebrovascular events last year |  |  |  |  |  |  |  | 0.881 |
| -  N-Miss | 0 | 2 | 2 | 1 | 0 | 0 | 5 |  |
| -  No | 12 (92.3%) | 21 (95.5%) | 18 (100.0%) | 34 (97.1%) | 14 (100.0%) | 13 (100.0%) | 112 (97.4%) |  |
| -  Yes | 1 (7.7%) | 1 (4.5%) | 0 (0.0%) | 1 (2.9%) | 0 (0.0%) | 0 (0.0%) | 3 (2.6%) |  |

## **Supplementary Table 2.**

Associations between each cluster and Cluster 4 (reference) resulted from the four multinomial regression models (sociodemographic, vascular, lifestyle, and medical). **(A)** Numerical values of Odds ratios (ORs) and 95% confidence intervals (CIs). Asterisks (*) and red colour indicate statistical significance. **(B)** Visual representation of OR (log transformed for visualization purposes) with 95% CI. Black dots indicate the OR and coloured lines indicate the CI of each cluster for each predictor.

|  | Cluster 1 | | | | Cluster 2 | | | | | | Cluster 3 | | | | Cluster 5 | | | | | Cluster 6 | | | | |
| --- | --- | --- | --- | --- | --- | --- | --- | --- | --- | --- | --- | --- | --- | --- | --- | --- | --- | --- | --- | --- | --- | --- | --- | --- |
|  | **OR** | | **95% CI** | | **OR** | | | **95% CI** | | | **OR** | | **95% CI** | | **OR** | | **95% CI** | | | **OR** | | **95% CI** | | |
| Sociodemographic characteristics | | | | | | | | | | | | | | | | | | | | | | | | |
| Age | 1.0 | 0.9 | | 1.3 | 1.0 | 0.9 | | | 1.1 | | 1.0 | 0.9 | | 1.2 | 1.1 | 0.9 | | 1.2 | 1.2 | | 1.0 | | 1.5 |  |
| Number of women | 0.1* | 0.0 | | 0.8 | 1.1 | 0.3 | | | 3.4 | | 0.5 | 0.1 | | 2.0 | 1.3 | 0.3 | | 6.4 | 0.5 | | 0.1 | | 3.0 |  |
| Years of education | 1.1 | 0.8 | | 1.7 | 0.9 | 0.7 | | | 1.2 | | 1.0 | 0.7 | | 1.3 | 0.9 | 0.6 | | 1.2 | 0.8 | | 0.5 | | 1.3 |  |
| Married or cohabiting | 0.7 | 0.1 | | 8.7 | 0.6 | 0.1 | | | 2.7 | | 0.3 | 0.1 | | 1.5 | 0.3 | 0.0 | | 1.7 | 0.9 | | 0.1 | | 11.8 |  |
| Vascular factors | | | | | | | | | | | | | | | | | | | | | | | | |
| Systolic blood pressure | 0.4* | 0.2 | | 0.9 | 1.1 | 0.6 | | | 1.9 | | 0.4* | 0.2 | | 0.9 | 1.1 | 0.5 | | 2.5 | 2.1 | | 0.8 | | 5.1 |  |
| Diastolic blood pressure | 2.6* | 1.1 | | 6.2 | 0.8 | 0.5 | | | 1.4 | | 2.5* | 1.3 | | 5.1 | 1.0 | 0.4 | | 2.2 | 0.6 | | 0.2 | | 1.4 |  |
| Total cholesterol | 0.3* | 0.2 | | 0.7 | 0.7 | 0.4 | | | 1.0 | | 0.8 | 0.4 | | 1.3 | 0.4* | 0.2 | | 0.8 | 1.6 | | 0.7 | | 3.4 |  |
| Glucose response after 2h | 2.9* | 1.4 | | 5.7 | 1.1 | 0.7 | | | 1.8 | | 1.8 | 1.0 | | 3.3 | 1.6 | 0.8 | | 3.2 | 1.3 | | 0.6 | | 2.6 |  |
| Obesity or overweight | 1.1 | 0.5 | | 2.3 | 1.2 | 0.7 | | | 1.9 | | 0.4* | 0.2 | | 0.8 | 1.5 | 0.7 | | 3.0 | 2.5* | | 1.2 | | 5.3 |  |
| Lifestyle | | | | | | | | | | | | | | | | | | | | | | | | |
| Lifestyle composite score | 0.8 | 0.4 | | 1.3 | 1.1 | 0.7 | | | 1.6 | | 0.7 | 0.4 | | 1.1 | 1.4 | 0.8 | | 2.7 | 1.1 | | 0.6 | | 2.1 |  |
| Self-reported medical conditions | | | | | | | | | | | | | | | | | | | | | | | | |
| Vascular conditions | 0.6 | 0.2 | | 1.7 | 1.3 | | 0.6 | | | 3.0 | 1.8 | 0.7 | | 4.3 | 0.5 | 0.2 | | 1.4 | 0.8 | | 0.3 | | 2.5 |  |
| Metabolic conditions | 1.2 | 0.6 | | 2.4 | 1.0 | | 0.5 | | | 1.7 | 1.1 | 0.6 | | 2.2 | 1.7 | 0.9 | | 3.3 | 1.1 | | 0.5 | | 2.6 |  |

## **Supplementary Table 3.**

β (beta estimates) of the four cognitive Generalized Linear Models (GLMs). Cluster 4 is set as reference for prediction measures. Asterisks (*) and red color indicate statistical significance. Points (.) indicate marginal significance.

|  | β (Estimate) | SE | t value | p-value |
| --- | --- | --- | --- | --- |
| Global cognition | | | | |
| Cluster 1 | -0.33 | 0.16 | -2.01 | 0.047 * |
| Cluster 2 | -0.02 | 0.13 | -0.15 | 0.880 |
| Cluster 3 | -0.25 | 0.14 | -1.79 | 0.075 |
| Cluster 5 | -0.29 | 0.16 | -1.81 | 0.073 |
| Cluster 6 | -0.47 | 0.16 | -2.90 | 0.005 ** |
| Executive functioning | | | | |
| Cluster 1 | -0.17 | 0.18 | -0.96 | 0.340 |
| Cluster 2 | 0.02 | 0.14 | 0.14 | 0.889 |
| Cluster 3 | -0.27 | 0.15 | -1.75 | 0.082 |
| Cluster 5 | -0.31 | 0.17 | -1.79 | 0.076 |
| Cluster 6 | -0.55 | 0.18 | -3.11 | 0.002 ** |
| Processing speed | | | | |
| Cluster 1 | -0.38 | 0.25 | -1.53 | 0.130 |
| Cluster 2 | -0.09 | 0.20 | -0.42 | 0.674 |
| Cluster 3 | -0.43 | 0.22 | -2.00 | 0.048 * |
| Cluster 5 | -0.24 | 0.24 | -0.98 | 0.329 |
| Cluster 6 | -0.61 | 0.25 | -2.41 | 0.017 * |
| Memory | | | | |
| Cluster 1 | -0.44 | 0.19 | -2.31 | 0.023 * |
| Cluster 2 | -0.03 | 0.15 | -0.17 | 0.864 |
| Cluster 3 | -0.15 | 0.16 | -0.93 | 0.355 |
| Cluster 5 | -0.28 | 0.18 | -1.51 | 0.134 |
| Cluster 6 | -0.33 | 0.19 | -1.72 | 0.089 |

**Supplementary Table 4.**
Sample sizes, minimum detectable effects (MDEs), and observed intervention effects for MRI cortical thickness and cognitive outcomes. MDEs assume 80% power for cluster-level analyses; overall sample power was 75–78% based on hierarchical mixed models. Observed effects (β or z-scores) with SE or p-values are shown.

| Analysis level | Sample size (Intervention vs Control) | Metric | MDE (80% power) | Observed effect (β / z-scores) | SE / p-value |
| --- | --- | --- | --- | --- | --- |
| Overall MRI sub-sample | 90 (47 vs 43) | β (MRI) | ~0.012 mm (*75% power*) | β = ±0.001 to 0.008 | NS |
|  |  | β (cognitive z-score) | ~0.299 SD (*78% power*) | z = 0.050 to 0.175 | NS |
| Cluster 4 | 31 (18 vs 13) | β (mean thickness, mm) | ~0.021 mm | Reference cluster | Reference cluster |
|  |  | β (cognitive z-score) | ~0.528 SD | Reference cluster | Reference cluster |
| Cluster 1 | 9 (3 vs 6) | β (mean thickness, mm) | ~0.046 mm | β = 0.003 to 0.075 | SE = 0.03 p = 0.006 |
|  |  | β (cognitive z-score) | ~1.156 SD | z = -0.192 to 0.415 | NS |
| Cluster 2 | 20 (6 vs 14) | β (mean thickness, mm) | ~ 0.029 mm | β = 0.032 to 0.051 | SE = 0.02 to 0.03 p = 0.023 to 0.044 |
|  |  | β (cognitive z-score) | ~0.723 SD | z = -0.056 to 0.203 | NS |
| Cluster 3 | 14 (5 vs 9) | β (mean thickness, mm) | ~0.034 mm | β = 0.046 to 0.067 | SE = 0.02 to 0.03 p = 0.015 to 0.037 |
|  |  | β (cognitive z-score) | ~0.851 SD | z = -0.293 to 0.206 | NS |
| Cluster 5 | 9 (6 vs 3) | β (mean thickness, mm) | ~0.046 mm | β = 0.006 to 0.037 | NS |
|  |  | β (cognitive z-score) | ~1.156 SD | z = -0.092 to 0.215 | NS |
| Cluster 6 | 7 (5 vs 2) | β (mean thickness, mm) | ~0.059 mm | β = -0.019 to 0.006 | NS |
|  |  | β (cognitive z-score) | ~1.470 SD | z = -0.621 to 0.502 | NS |
